# Inactivation of RASA1 promotes melanoma tumorigenesis via R-Ras activation

**DOI:** 10.18632/oncotarget.8127

**Published:** 2016-03-16

**Authors:** Hyeran Sung, Krishna L. Kanchi, Xue Wang, Kristen S. Hill, Jane L. Messina, Ji-Hyun Lee, Youngchul Kim, Nathan D. Dees, Li Ding, Jamie K. Teer, Shengyu Yang, Amod A. Sarnaik, Vernon K. Sondak, James J. Mulé, Richard K. Wilson, Jeffrey S. Weber, Minjung Kim

**Affiliations:** ^1^ Department of Molecular Oncology, Moffitt Cancer Center, Tampa, FL, USA; ^2^ Comprehensive Melanoma Research Center, Moffitt Cancer Center, Tampa, FL, USA; ^3^ The Genome Institute, Washington University, St. Louis, MO, USA; ^4^ Department of Cutaneous Oncology, Moffitt Cancer Center, Tampa, FL, USA; ^5^ Department of Pathology, Moffitt Cancer Center, Tampa, FL, USA; ^6^ Department of Internal Medicine, University of New Mexico Comprehensive Cancer Center, Albuquerque, NM, USA; ^7^ Department of Biostatistics and Bioinformatics, Moffitt Cancer Center, Tampa, FL, USA; ^8^ Department of Medicine, Washington University, St. Louis, MO, USA; ^9^ Department of Genetics, Washington University, St. Louis, MO, USA; ^10^ Department of Tumor Biology, Moffitt Cancer Center, Tampa, FL, USA; ^11^ Department of Medicine, NYU Langone Medical Center, New York, NY, USA

**Keywords:** melanoma, RASA1, RasGAP, R-Ras, whole genome sequencing

## Abstract

Inactivation of Ras GTPase activating proteins (RasGAPs) can activate Ras, increasing the risk for tumor development. Utilizing a melanoma whole genome sequencing (WGS) data from 13 patients, we identified two novel, clustered somatic missense mutations (Y472H and L481F) in *RASA1* (RAS p21 protein activator 1, also called p120RasGAP). We have shown that wild type RASA1, but not identified mutants, suppresses soft agar colony formation and tumor growth of *BRAF* mutated melanoma cell lines via its RasGAP activity toward R-Ras (related RAS viral (r-ras) oncogene homolog) isoform. Moreover, R-Ras increased and RASA1 suppressed Ral-A activation among Ras downstream effectors. In addition to mutations, loss of RASA1 expression was frequently observed in metastatic melanoma samples on melanoma tissue microarray (TMA) and a low level of *RASA1* mRNA expression was associated with decreased overall survival in melanoma patients with *BRAF* mutations. Thus, these data support that RASA1 is inactivated by mutation or by suppressed expression in melanoma and that RASA1 plays a tumor suppressive role by inhibiting R-Ras, a previously less appreciated member of the Ras small GTPases.

## INTRODUCTION

Ras is a small GTP binding protein that is frequently mutated in human melanoma. Although mutations in *KRAS* (2%) and *HRAS* (1%) have been observed in melanoma specimens, *NRAS* is the most commonly mutated Ras family member (15~20%) among the three closely related classical Ras proteins [1, 2]. While mutant Ras (primarily at residues 12, 13, or 61) is locked in an active GTP-bound form, wild type RAS cycles between inactive GDP- and active GTP-bound states, which can be regulated by activity of Ras GTPase activating proteins (RasGAPs) and Ras guanine nucleotide exchange factors (RasGEFs) [3]. RasGAPs mediate inactivation of RAS proteins by enhancing the weak intrinsic GTPase activity of RAS; thus, inactivation of RasGAPs may increase the risk for tumor development. Currently, various RasGAPs with overlapping patterns of tissue distribution but with nonredundant functions have been identified, including *RASA1, NF1*, the GAP1 family (*RASA2, RASA3, RASA4, RASAL1*), and the SynGAP family (*DAB2IP, RASAL2, RASAL3, SynGAP*) as well as IQGAPs and Plexin receptors [4, 5]. Tumor suppressive function of *NF1* (neurofibromatosis type 1) in several cancer types including melanoma [6–8], *DAB2IP* in prostate cancer [9], *RASAL2* in breast cancer [10], *PLXNC1* (Plexin C1) [11] and *RASA2* [12] in melanoma have been described.

RASA1 (RAS p21 protein activator (GTPase activating protein)), also called p120RasGAP, is the first identified RasGAP protein. In addition to the RasGAP domain, RASA1 contains 2 SH2 (Src homology 2) domains, a SH3 (Src homology 3) domain, a PH (Pleckstrin homology) domain, and a C2 domain (Calcium-dependent phospholipid binding domain), and interacts with important signaling molecules as a signaling scaffold protein [13, 14]. RASA1 has been implicated in many biological processes including actin filament polymerization, vascular development, cellular apoptosis, and cell motility [15, 16]. Mice null for *Rasa1* showed abnormal blood vessel growth, extensive neuronal apoptosis, and embryonic death at E10.5 [17]. Loss of *Rasa1* in endothelial cells led to increased endothelial proliferation and tube formation [18]. *RASA1* germline mutations in humans have been linked to capillary malformation-arteriovenous malformation (CM-AVM), an autosomal dominant disorder characterized by atypical capillary malformations [19]. Asides from these physiological roles of RASA1, its importance in tumorigenesis, particularly in melanoma, has not been addressed previously.

Recently, we analyzed 15 melanoma genomes and matched normal genomes from peripheral blood mononuclear cells (PBMC) from 13 patients by high-throughput whole genome sequencing and identified a large number of novel genetic alterations of melanoma [20]. Consistent with the importance of the Ras-Raf-MAPK pathway activation in melanoma, many somatic missense mutations in genes involved in or regulating this pathway including *NRAS*, *BRAF, MAPKs*, and RasGAPs have been identified. In this study, we focused on *RASA1*, which featured two novel, clustered somatic missense mutations (Y472H and L481F) and sought to determine the function of RASA1 in melanoma tumorigenesis. Through gain of function and loss of function studies, we have shown that RASA1 plays a tumor suppressive role in melanoma. Additionally, the identified mutations in *RASA1* have functional impacts in that the Y472H mutation promoted tumor growth and the L481F mutation down-modulated a tumor suppressive role. We also have shown that RASA1 functions as a RasGAP for the R-Ras isoform. In addition, we addressed the expression pattern of RASA1 in a melanoma TMA containing dysplastic nevi, primary, and lymph node and distant metastatic melanomas and observed frequent RASA1 down-regulation in metastatic melanomas.

## RESULTS

### Melanoma genome sequencing uncovers clustered novel somatic mutations in *RASA1*

**Figure 1:**
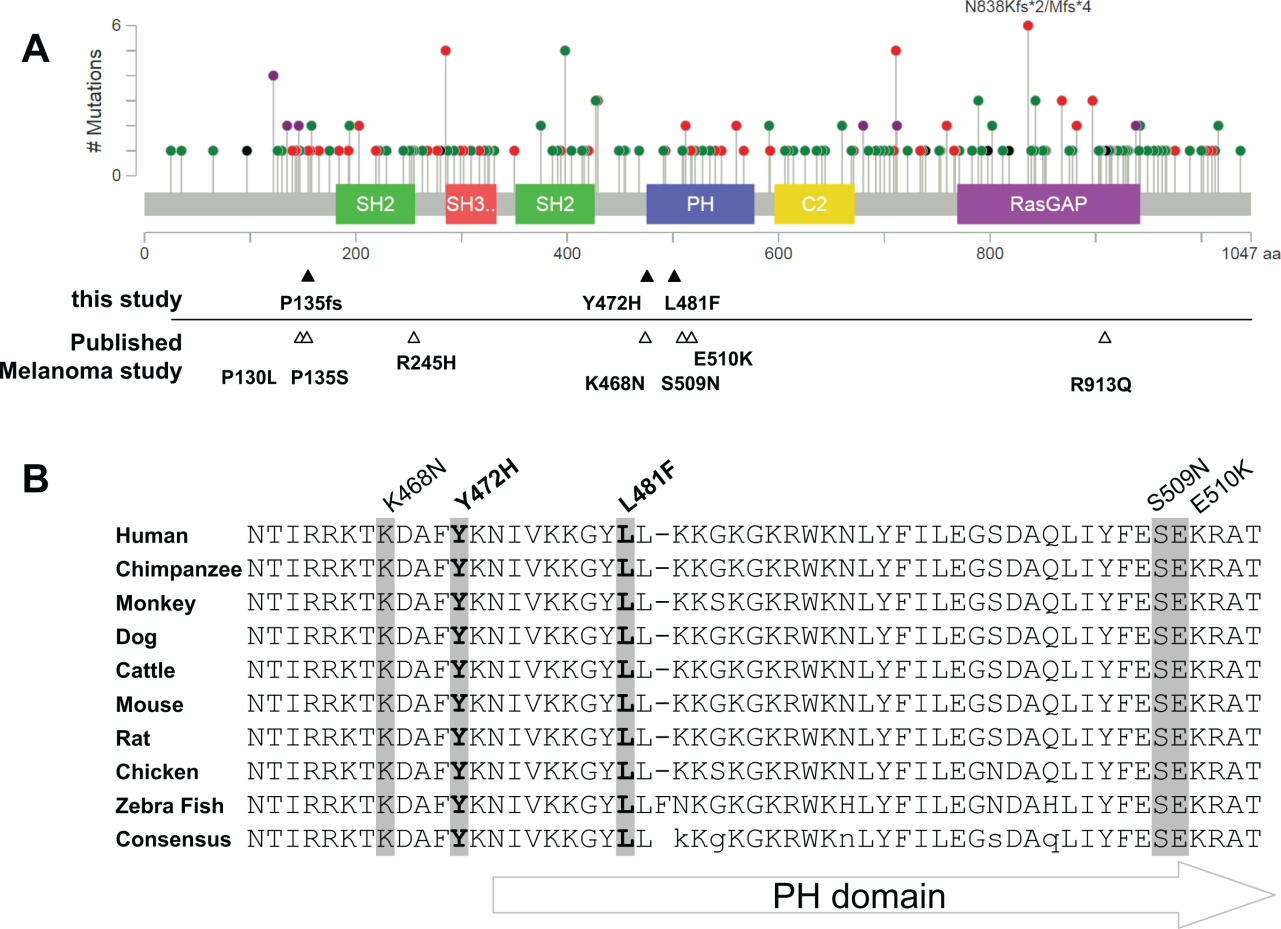
Mutation distribution in *RASA1* (**A**) A schematic of human RASA1 showing the distribution of the mutations identified in all cancer types (adapted from cBioPortal) with conserved protein functional domains: SH2, PH, SH3, C2, and RasGAP domains. Green: missense mutation; Red: nonsense, frame shift, and splice site mutation; Black: in frame deletion; and Purple: combination of Red and Green mutations. Note some recurrent alterations. Mutations identified in melanomas are marked under the graph (filled triangles: this study, empty triangles: published melanoma studies). (**B**) Alignment of RASA1 sequence around the PH domain among different species. Five out of ten mutations identified in melanomas are localized within this highly conserved region (< 50 amino acids).

Our WGS analysis of 15 metastatic melanoma genomes and matched normal PBMC genomes from 13 patients and subsequent targeted exome sequencing of an additional 15 normal and melanoma pairs revealed a large number of complex alterations [20]. Mutation proximity analysis of these data sets using MuSiC [21] identified mutation clusters in 175 genes such as *BRAF, NRAS, MAPK10*, and *RASA1* as a possible indication for positive selection (Supplementary Table S1). Among the components of the Ras-Raf-MAPK pathway with novel mutation clusters, we focused on *RASA1* with two neighboring somatic missense mutations, p.Tyr472His (Y472H) and p.Leu481Phe (L481F) as a novel candidate that may dysregulate the Ras pathway. In our extension screening involving an additional 97 melanoma samples from 96 patients, a frame shift deletion in *RASA1* was observed affecting P135 (Figure 1A). To expand upon these findings, we searched publically available databases for *RASA1* alterations. Large scale melanoma genomic studies listed in cBioPortal [22, 23] have shown somatic missense mutations of *RASA1* in 2 out of 121 patients (P135S and E510K) [24], 1 of 91 (R245H) [8], and 3 of 228 (P130L, K468N, S509N, and R913Q) [25] (Figure 1A). Thus, genetic alterations of *RASA1* occur in melanoma, although the frequency is low. Interestingly, *RASA1* alterations in melanoma are significantly more clustered in or around the PH domain than expected by chance (Randomization test, *p-*value < 0.001) and target highly conserved amino acids among different species (Figure 1 and Supplementary Figure S1). This suggests that some of these clustered mutations may function as gain-of-function or dominant negative mutations, as observed in p53 [26] and in PTEN [27].

### RASA1 is down-regulated in human metastatic melanoma samples

**Figure 2:**
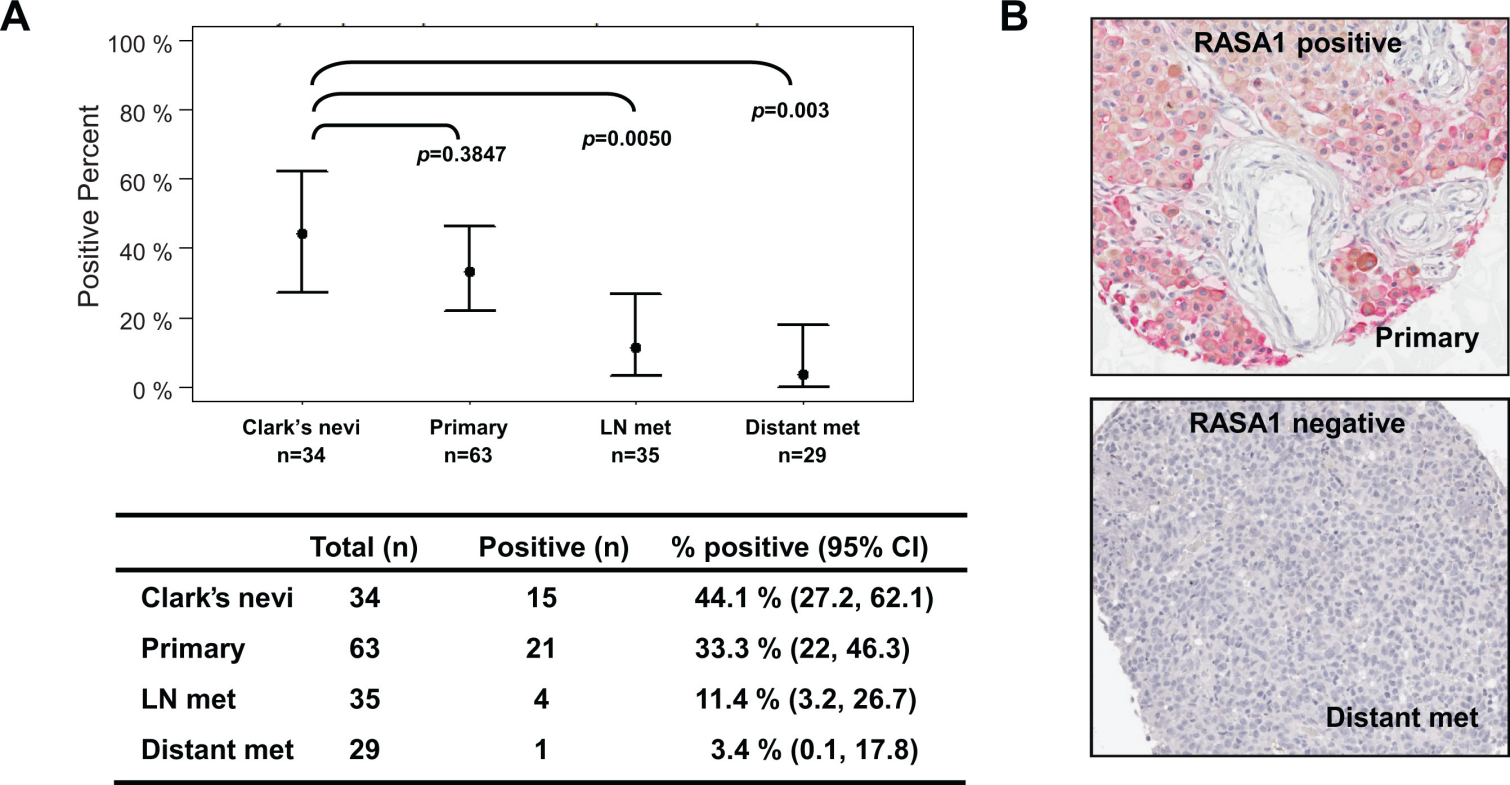
RASA1 is down-regulated in metastatic melanomas RASA1 protein level on a human melanoma tissue microarray (TMA) containing Clark's nevi (*n* = 34), primary melanomas (*n* = 63), lymph node metastases (*n* = 35) and distant metastases (*n* = 29). In (**A**) the number of samples with RASA1 expression in each category and confidence interval (CI) with associated *p-*values comparing to Clark's nevi group (Bonferroni corrected Fisher's exact test) is shown. Representative images of RASA1 positive and negative cores are shown in (**B**).

Tumor suppressor genes can also be inactivated by loss of expression as well as by mutation and deletion. Since *RASA1* mutation rate was low in melanoma, we determined the expression level of RASA1 in melanocytic lesions *in vivo* by immunohistochemical analysis on a melanoma TMA containing atypical (Clark's) nevi, primary melanomas, lymph node metastases, and distant metastases. Positive RASA1 staining was observed in 44.1% (15/34) of Clark's nevi, 33.3% (21/63) of primaries, 11.4% (4/35) of lymph node metastases, and 3.4% (1/29) of distant metastases (Figure 2). Therefore, RASA1 expression was frequently down-regulated in metastatic melanoma samples compared to primary melanomas and atypical nevi.

### Low level of *RASA1* mRNA expression is associated with decreased overall survival of melanoma patients with *BRAF* mutation

**Figure 3:**
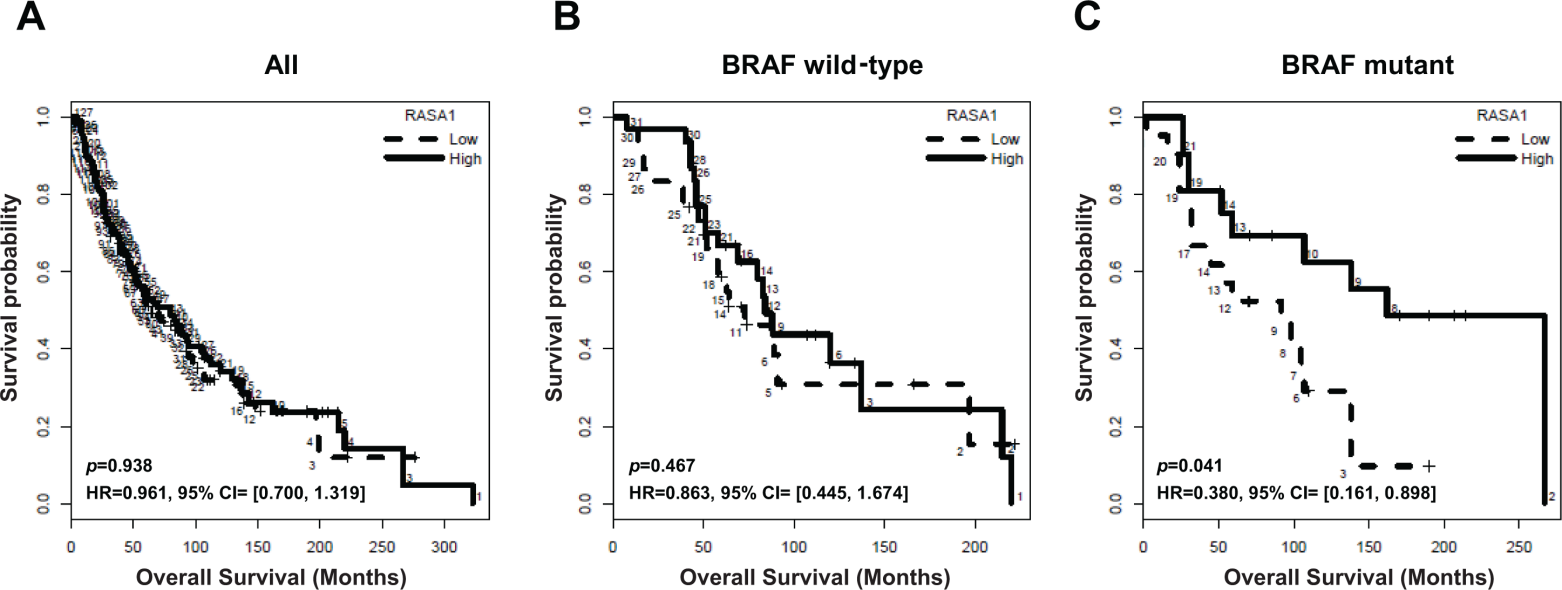
Association of *RASA1* mRNA level with overall survival of melanoma patients Kaplan-Meier overall survival curves of melanoma patients in this cohort for all samples (**A**) (*n* = 253), *BRAF* wild type (**B**) (*n* = 61), and *BRAF* mutant samples (**C**) (*n* = 42) are shown. Samples are divided into high (above median) and low (below median) *RASA1* expression groups, based on the average intensity of the two *RASA1* probe sets.

To address whether RASA1 expression is associated with clinical outcome of melanoma patients, we examined the association between RASA1 expression level and overall survival in a historical cohort of 253 metastatic melanoma samples collected at the Moffitt Cancer Center under the Total Cancer Care (TCC) project. In all samples, there was no significant association between *RASA1* expression (average intensity of the two *RASA1* probe sets) and overall survival (Figure 3A; *p =* 0.938, HR = 0.961; 95% CI, 0.7 to 1.319). Targeted exome sequencing data was available for 106 out of these 253 samples. Next, we divided these samples based on *BRAF* mutation status following exclusion of samples with *RASA1* mutation from 3 patients. In *BRAF* wild type group (*n* = 61), *RASA1* level was not associated with overall survival (Figure 3B; *p =* 0.467, HR = 0.863; 95% CI, 0.445 to 1.674). However, in *BRAF* mutant group (*n* = 42), overall survival of two groups stratified based on *RASA1* expression level was significantly different (Figure 3C; *p =* 0.041, HR = 0.38; 95% CI, 0.161 to 0.898). This data suggests that a low level of *RASA1* expression is associated with poorer overall survival of patients harboring *BRAF* mutations and that *RASA1* deficiency may cooperates with *BRAF* mutation in melanoma tumorigenesis. A summary of the samples included in this analysis are provided in Supplementary Table S2 and survival curves for individual probe sets are shown in Supplementary Figure S2B and S2C.

### Loss of RASA1 enhances anchorage-independent growth and subcutaneous tumor growth

**Figure 4:**
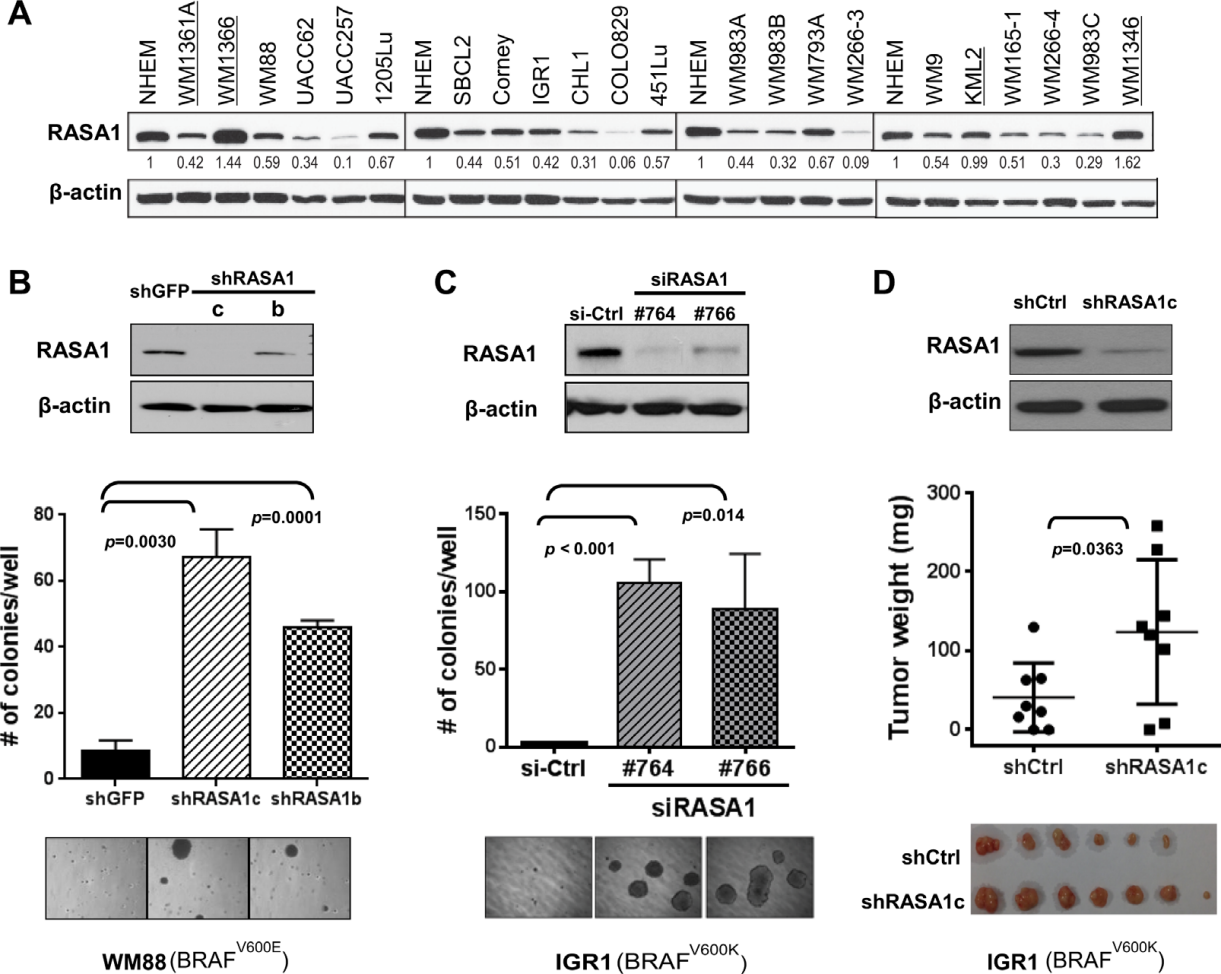
Loss of RASA1 promotes anchorage-independent colony formation (**A**) RASA1 expression in human melanoma cell lines in comparison to normal human epithelial melanocytes (NHEM). Protein level was quantitated using image J software and relative RASA1 to β-actin ratio was normalized against that of NHEM. Cell lines with *NRAS* mutation are underlined. (**B** and **C**) (Top) Immunoblotting of RASA1 protein levels in WM88 transduced with shRNA targeting *RASA1* (shRASA1c and b) or shGFP (B) and in IGR1 cells transfected with siRNA targeting *RASA1* (si#764and #766) or siCtrl (non-targeting siRNA) on day 4 post transfection (C). (Bottom) Number of colonies grown on soft agar on day 21 (B) or on day 15 (C) counted per well. Graph shows data as mean +/− SD. Representative microscopic images are shown. (**D**) Subcutaneous tumors generated with IGR1 cells with pLKO.1 vector control (shCtrl) or with shRASA1c (bottom) and tumor weights (middle) on day 48 for each group. Data expressed as mean +/− SD.

In order to address RASA1 function in melanoma, we first evaluated RASA1 protein levels in human melanoma cell lines. RASA1 levels were over 50% down-regulated in 12 out of 21 (57%) human melanoma cells compared to a normal human epithelial melanocyte line (NHEM) (Figure 4A). Both cell lines with increased RASA1 expression (WM1366 and WM1346) contained activating *NRAS* mutations. RasGAPs can inactivate wild type but not mutant Ras proteins and low level of *RASA1* mRNA expression is associated with decreased overall survival of melanoma patients with a *BRAF* mutation; therefore, we addressed RASA1 function in melanoma cell lines containing *BRAF* mutations.

First, we examined the effect of RNAi-mediated suppression of RASA1 function in melanoma cell lines on anchorage-independent soft agar colony formation. Decreased *RASA1* expression via shRNAs (shRASA1c and b) in WM88 increased anchorage-independent growth (7.7 fold (*p =* 0.003) and 5.3 fold (*p* < 0.001), respectively) on day 21 compared to control cells (Figure 4B). A modest increase in proliferation by *RASA1* knock-down was also observed in WM88 (data not shown). In addition, compared to control cells with non-targeting siRNA, IGR1 cells with #764 and #766 siRNAs targeting *RASA1* showed decreased RASA1 protein expression and significantly increased colony formation (*p* < 0.001 and *p =* 0.014, respectively) (Figure 4C). Knock-down of *RASA1* in IGR1 cells did not enhance proliferation or invasion through matrigel in a Boyden chamber assay (data not shown). When injected subcutaneously into nude mice, IGR1 cells with shRASA1c showed increased tumor growth compared to control cells with empty vector (Figure 4D and Supplementary Figure S3A). On day 48, the average tumor weight was 40.9 ± 15.5 mg (mean ± SEM) for control group compared to 123.9 ± 32.2 mg for shRASA1c. This is a statistically significant difference (*p =* 0.036). Thus, loss of RASA1 expression promotes anchorage-independent colony formation and tumor growth, supporting that RASA1 plays a tumor suppressive role in melanoma.

### Wild type, but not mutant, RASA1 suppresses anchorage-independent colony formation and subcutaneous tumor growth

**Figure 5:**
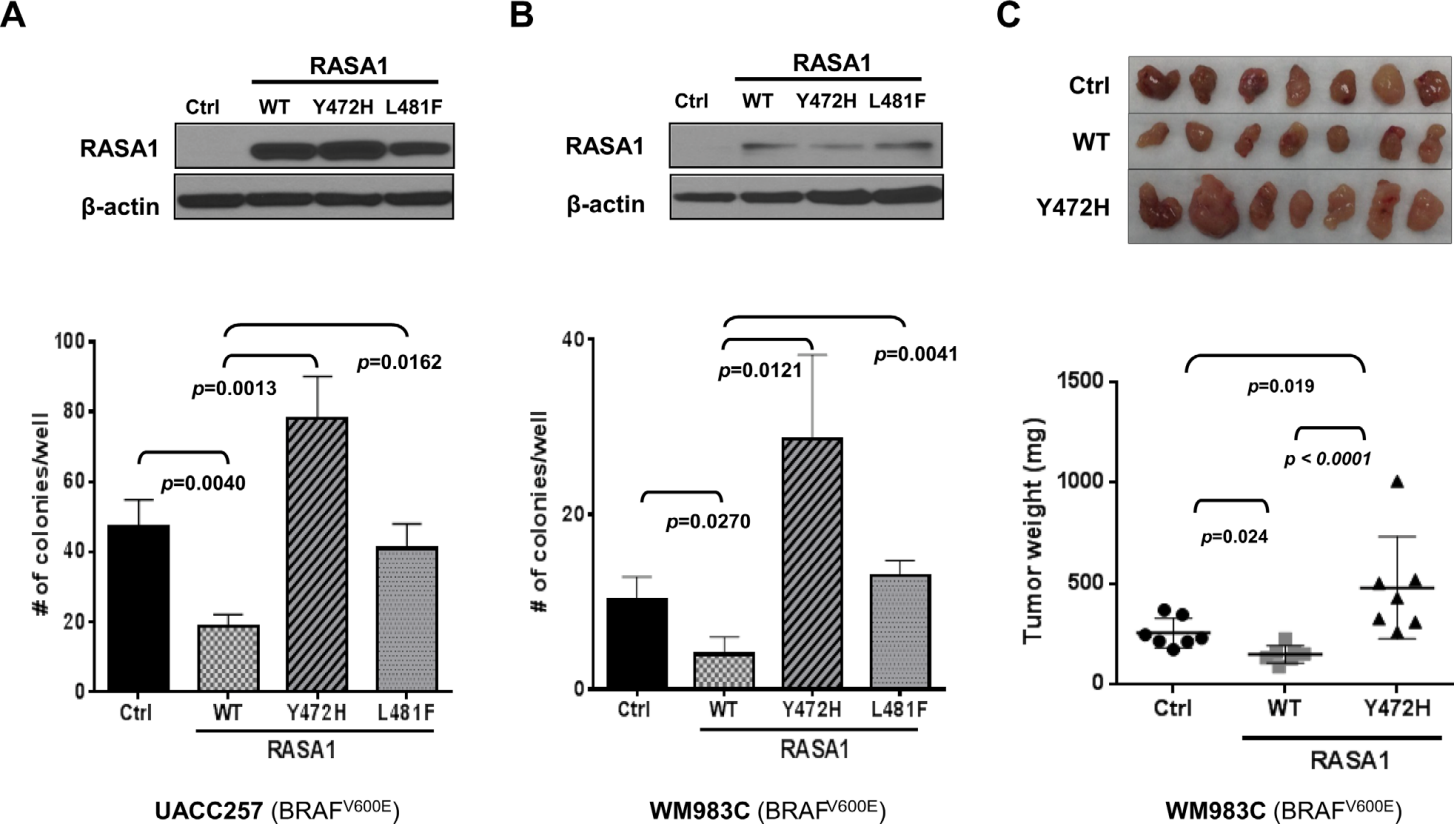
Expression of RASA1 wild type (WT), but not Y472H and L481F mutants, suppresses anchorage-independent growth *in vitro* and tumor growth *in vivo* Wild type (WT), Y472H, or L481F mutant form of RASA1 was expressed in UACC257 (**A**) and WM983C (**B** and **C**). (A and B) Ectopic expression was confirmed by immunoblotting with anti-RASA1 antibody (top). Graph shows the mean (+/− SD) number of colonies grown on soft agar on day 21 counted per well (Bottom). (C) Subcutaneous tumors generated with WM983C cells (top) and tumor weights (bottom) on day 29 for each group. Statistical analysis was performed using one-way ANOVA with Tukey's multiple comparison adjustment. Data shown graphically as mean +/− SD.

When RASA1 was expressed in UACC257 and WM983C melanoma cells with low endogenous RASA1 expression, decreased soft agar colony formation was observed compared to controls with empty vector (Figure 5A and 5B), consistent with a tumor suppressive role for RASA1. We next evaluated the effects of the identified RASA1 mutations on anchorage-independent growth. The L481F mutation rendered RASA1 defective in suppressing anchorage-independent colony growth both in UACC257 and WM983C. Interestingly, the RASA1 Y472H mutant promoted colony growth in these cells, converting RASA1 from a tumor suppressor to a putative oncogene. Together, these loss- and gain-of function studies suggest that RASA1 plays a tumor suppressive role in melanoma by restraining anchorage-independent growth and RASA1 Y472H and L481F missense mutations are pathogenic alterations that promote colony formation or remove its tumor suppressive function, respectively. Similar observations were made in SKmel28 and in M14 (Supplementary Figure S4).

To determine whether RASA1 can suppress tumor growth *in vivo*, we injected WM983C cells harboring a vector control, RASA1 wild type, or RASA1 Y472H mutant into nude mice subcutaneously (*n* = 7 each). Tumors developed first in mice implanted with RASA1 Y472H cells, followed by those with control and with RASA1 wild type (Supplementary Figure S3B). The average weight of tumors on day 29 was 149.4 ± 16.12 mg (mean ± SEM, *p =* 0.024) for RASA1 wild type and 479.3 ± 95.63 mg (*p =* 0.019) for Y472H compared to 256.0 ± 27.63 mg for control (Figure 5C). Therefore, tumor growth in mice is inhibited by wild type RASA1, but it is enhanced by RASA1 Y472H mutant, which is consistent with their effect on soft agar colony formation *in vitro*. This result supports that RASA1 suppresses melanoma growth.

### RASA1 functions via its effects on Ras

**Figure 6:**
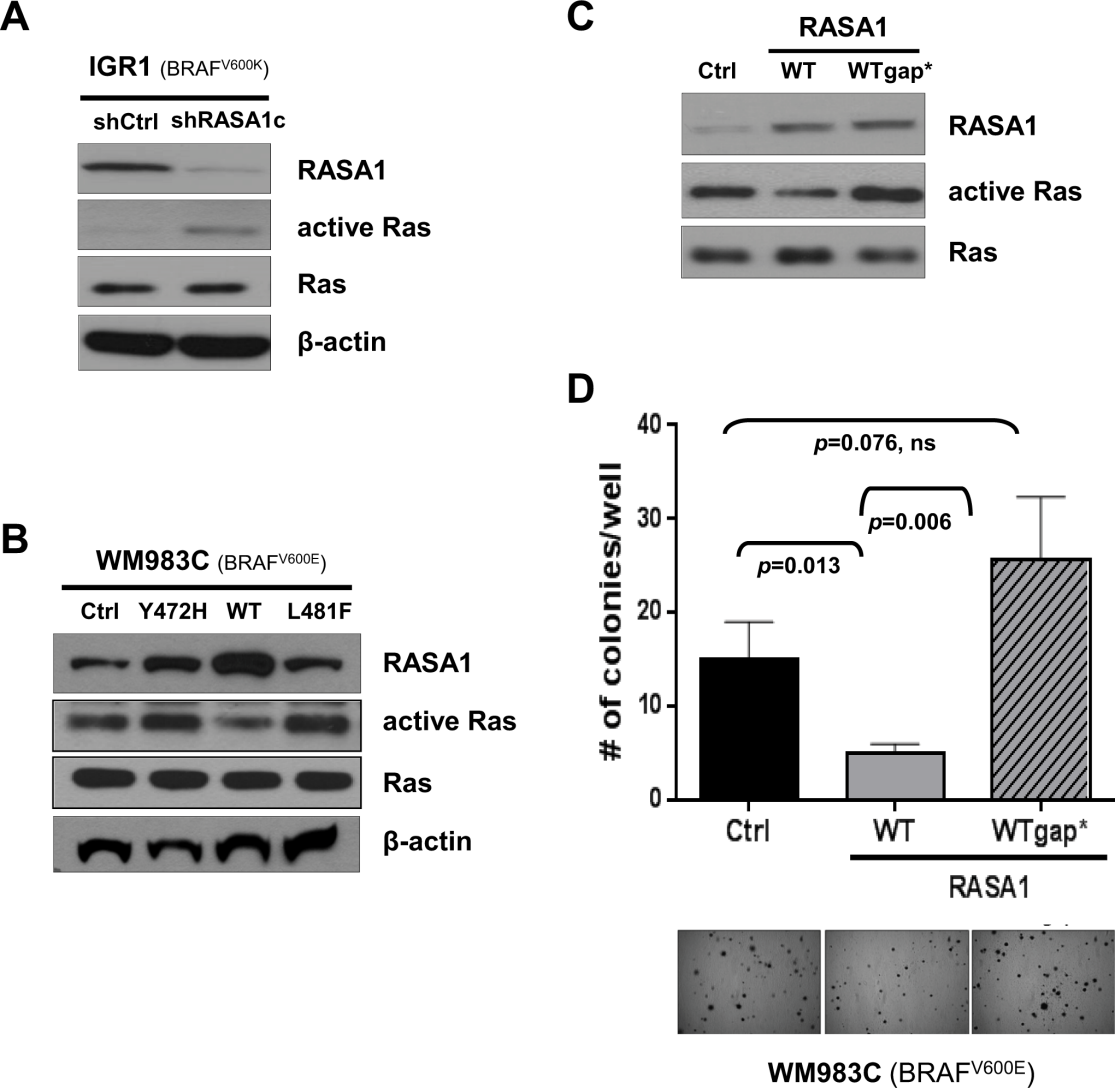
RASA1 functions as a tumor suppressor via its effects on Ras (**A** and **B**) Ras activity in IGR1 cells with or without siRNA-mediated *RASA1* knock-down (A) and in WM983C cells with vector control (Ctrl) or with wild type (WT), Y472H, or L481F mutant forms of RASA1 expression (B) was determined by pulling down active GTP-bound Ras and immunoblotting with pan Ras antibody. (**C** and **D**) Ras activity of WM983C cells expressing wild type RASA1 (WT) or Q938H mutant RASA1 (WTgap*) with impaired RasGAP activity (C) and number of soft-agar colonies per well on day 21 (mean +/− SD) with representative microscopic images (D).

Although RASA1 is a RasGAP, RASA1 also has Ras-independent function through its SH2-SH3-SH2-PH domains [4, 13, 14]. To understand molecular mechanisms underlying RASA1 function, we first examined whether RASA1 modulates Ras activity in melanoma cells. GTP-bound active Ras was increased by RASA1 ablation via shRASA1c in IGR1 and was decreased by expression of wild type RASA1, but not of Y472H and L481F mutants, in WM983C (Figure 6A and 6B). When a RASA1 p.Gln938His (Q938H) mutant with impaired RasGAP activity [28] was expressed in WM983C, anchorage-independent colony formation was rescued, supporting the importance of its RasGAP function for tumor suppression (Figure 6C and 6D). Therefore, at least in part, RasGAP activity of RASA1 underlies its tumor suppressive function in melanoma.

### RASA1 suppresses activation of R-Ras and Ral-A

**Figure 7:**
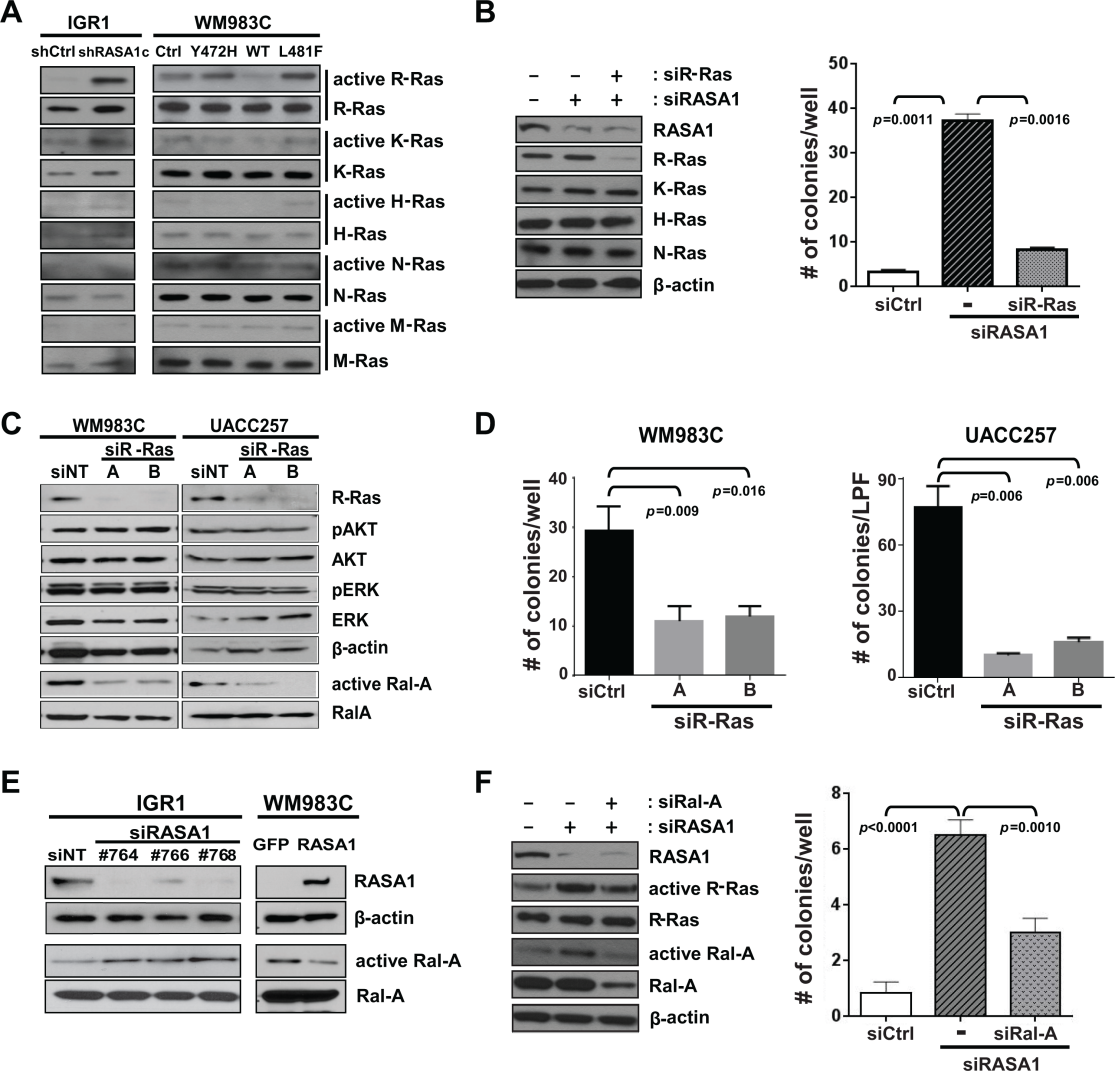
RASA1 suppresses activation of R-Ras and Ral-A (**A**) GTP-bound active-Ras in IGR1 and in WM983C is probed with isoform-specific antibodies against H-, K-, N-, M-, and R-Ras. (**B**) R-Ras expression is knocked-down with *RRAS* siRNA in IGR1 cells with RASA1 knock-down (#764 siRNA) (left: western blot). Number of soft-agar colonies per well expressed as mean +/− SD (right panel) on day 17 counted under the microscope are shown. (**C**) Level of R-Ras, pAKT, AKT, pERK, ERK, Ral-A and β-actin was determined by western blot analysis in WM983C and UACC257 cells with (A or B) or without (siNT) siRNA-mediated *R-Ras* knock-down. Ral-A activity was measured by pulling down GTP bound active Ral proteins with RalBP1-RBD agarose followed by immunoblotting with α-Ral-A antibody. (**D**) Number of soft-agar colonies per well for WM983C and per low power field (LPF) for UACC257 on day 21 per well (mean +/− SD). (**E**) Level of RASA1, β-actin, and Ral-A and active GTP-bound Ral-A was determined by western blot analysis in IGR1 with (#764, 766, or 768) or without (siNT) siRNA-mediated *RASA1* knock-down and in WM983C cells transduced with adenovirus encoding RASA1 or GFP. (**F**) Number of soft-agar colonies visible to the naked eye were counted on day 12 per well (right) shown as mean +/− SD.

Distinct RasGAPs have differential substrate specificity toward each Ras isoforms. While NF1 shows higher activity toward K- and H-, but not N-Ras in melanoma [6], RASA1 has increased activity toward R-Ras compared with H-Ras [4, 29]. In order to identify Ras isoforms that are regulated by RASA1 in melanoma, GTP-bound active Ras was probed with isoform specific antibodies against H-, K-, N-, M-, and R-Ras. RASA1 knock-down in IGR1 significantly activated R-Ras. Moreover, expression of wild type RASA1, but not of Y472H and L481F mutants, in WM983C significantly inactivated R-Ras (Figure 7A). While RASA1 shows modest activity toward K-Ras as well, no change in active H-, N-, and M-Ras isoforms was observed (Figure 7A). Reduced RASA1 expression in M14 also activated, while increased RASA1 expression in UACC257 suppressed, R-Ras activation (Supplementary Figure S5A). Importantly, when R-Ras expression is knocked-down via *RRAS* siRNA in IGR1, enhanced colony formation by loss of RASA1 was suppressed (Figure 7B and Supplementary Figure S5B). This data suggests that R-Ras is required for colony formation driven by RASA1 inactivation, although involvement of other Ras isoforms cannot be ruled out.

To determine which potential R-Ras effectors are regulated downstream of RASA1, we first used two siRNAs (A and B) targeting R-Ras in WM983C and UACC257 and investigated three major downstream effectors of Ras signaling, ERK, AKT and Ral-A. When R-Ras expression is suppressed, active GTP-bound Ral-A was significantly reduced while phosphorylation of AKT or ERK was not significantly decreased (Figure 7C). Additionally, reduced R-Ras expression significantly suppressed colony growth (Figure 7D).

Having shown that RASA1 regulates R-Ras activity and that R-Ras regulates Ral-A activity in *BRAF* mutant melanoma, we next addressed if Ral-A activity was stimulated or suppressed by RASA1 knockdown or overexpression, respectively. Ral-A was activated by decreased RASA1 with 3 independent siRNAs targeting RASA1 (#764, 766, or 768) in IGR1 and M14 and suppressed by expression of wild type RASA1 in WM983C and UACC257 (Figure 7E and Supplementary Figure S6). Moreover, re-expression of RASA1 in M14 cells with siRNA targeting RASA1 suppressed Ral-A activation driven by reduced RASA1 expression (Supplementary Figure S6C). Neither reduced RASA1 expression in IGR1 and M14 nor over-expression of RASA1 in WM983C and UACC257 caused consistent alterations in AKT or ERK phosphorylation (data not shown). Therefore, RASA1 consistently suppresses Ral-A activation in these BRAF activated melanoma cell lines. In addition, when Ral-A expression is knocked-down via *RALA* siRNA in IGR1, colony formation induced by loss of RASA1 was suppressed (Figure 7F). Taken together, our data suggests that RASA1 suppresses R-Ras activation, thus preventing the activation of Ral-A.

## DISCUSSION

RASA1 is the first identified RasGAP that is ubiquitously expressed across different tissue types. Deletions and nonsynonymous mutations (including many truncating mutations; Figure 1A) of *RASA1* are frequently observed in many large-scale integrative cancer studies listed in cBioPortal, such as 13% in metastatic castration-resistant prostate adenocarcinoma [30], 8.8% in uterine endometrial carcinoma [31], and 6.7% in lung squamous cell carcinoma [32]. Although this suggests that RASA1 may play tumor suppressive roles in many cancer types, the functional impact of these mutations as well as the role of RASA1 in tumorigenesis has not been previously addressed in detail. Here, we have shown that RASA1 is inactivated by mutation or, more frequently, by decreased expression in melanoma and that RASA1 suppresses anchorage-independent growth *in vitro* and tumor growth *in vivo*. Our study supports that RASA1 suppresses melanoma growth at least in part by regulating R-Ras activity in melanoma cells harboring oncogenic *BRAF* mutations. Additional study is needed to reveal a possible broader role for RASA1 inactivation in other cancer types.

Each of the RasGAPs has differential substrate specificity toward Ras isoforms. Loss of NF1, RASAL2, and DAB2IP led to H- and K-Ras activation followed by ERK and AKT activation [6, 9, 10]. Loss of PLXNC1 (Plexin C1) activated R-Ras and AKT but not ERK [11]. RASA1 showed greater activity toward R-Ras than toward H-, N-, and K-Ras [29], which was consistent with our observations. Although R-Ras shares approximately 55% sequence identity with H-Ras, R-Ras is shown to preferentially activate PI3K and RalGEF, not RAF1 [33], suggesting isoform-specific functions. Interestingly, it is shown that R-Ras is expressed in 52% of primary melanomas compared to 4% of nevi and 14% of metastatic melanoma [11]. Although not frequently mutated, it is possible that R-Ras activation via inactivation of RASA1 may play important roles in melanoma.

**Figure 8:**
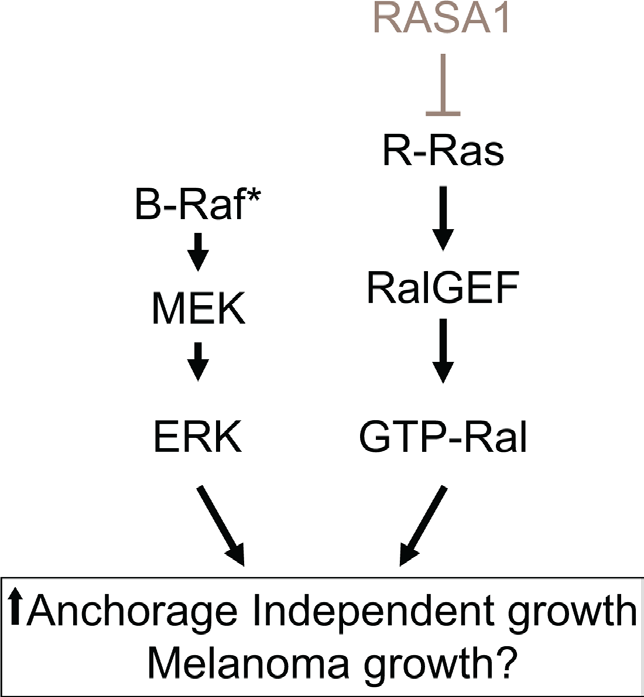
Model showing the putative cooperation of BRAF/MEK/ERK and RASA1/R-Ras/Ral-A pathways RASA1 inactivation by mutation or by reduced expression may cooperates with mutant BRAF by increasing R-Ras/Ral signaling to increase anchorage independent growth *in vitro* and xenograft tumor growth *in vivo*.

Previously, it was suggested that Ral activation may contribute to melanoma tumorigenesis [34] and that Ral-A is frequently activated in human melanoma cell lines independent of *NRAS* mutations [35]. In our study, loss of RASA1 triggered activation of R-Ras and Ral-A and R-Ras was required to drive colony formation driven by RASA1 inactivation in melanoma cells with *BRAF* mutation (Figure 7B). Moreover, we observed that R-Ras suppression reduced Ral-A activation and colony growth. Therefore, our study suggests that RASA1 inactivation and subsequent R-Ras activation is one of the possible mechanisms leading to Ral-A activation in melanomas with *BRAF* mutation (Figure 8). Possible cooperative interactions between activation of BRAF/MEK/ERK and RASA1/R-Ras/Ral-A pathways may explain the observed decreased overall survival of melanoma patients, who have low levels of RASA1 expression and *BRAF* mutations (Figure 3 and Figure 8). Our observation is consistent with a recent report that loss of a RasGAP, *Nf1*, cooperates with BRAF activation in melanoma tumorigenesis in a genetically engineered mouse model [6].

In melanoma cells harboring activating *NRAS* mutation, RASA1 may play differential roles as a signaling adaptor/scaffold protein, since RASA1 cannot function as a RasGAP on mutated Ras isoforms. In fact, it has been shown that RASA1 mediates the interaction between oncogenic Ras and c-Src, leading to c-Src activation and invasion in cells with Ras mutation [36]. Loss of RASA1 expression or RASA1 over-expression in human melanoma cell lines WM1366 and WM1361A harboring *NRAS* mutations (Q61L and Q61K, respectively) did not increase or decrease Ras activity or anchorage-independent growth, respectively (Supplementary Figure S7 and data not shown). Further study is required to determine the role of RASA1 in melanomas with oncogenic *NRAS* mutations, especially in metastasis, and the effect of RASA1 on R-Ras activation in melanomas that are wild-type for BRAF and NRAS.

In summary, RASA1 plays tumor suppressive roles in melanomas with BRAF activation. RASA1 functions as a RasGAP for the R-Ras isoform, for which a role has not been appreciated previously in melanoma tumorigenesis. R-Ras activation and subsequent Ral-A activation via inactivation of RASA1 may provide an additional mechanism driving melanoma formation. This study is the first demonstration of the role of RASA1 in the development of melanoma.

## MATERIALS AND METHODS

### Antibodies, reagents, and immunoblotting

Antibodies against β-actin and K-Ras were obtained from Sigma, H-Ras (c-20) and N-Ras (F155) from Santa Cruz Biotechnology, Ras and Ral-A from Upstate, M-Ras from Thermo Scientifics, R-Ras, AKT, p-AKT (S473), ERK1/2, p-ERK1/2 (T202/T204) from Cell Signaling Technology, and RASA1 from Epitomics. Active Ras-GTP and Ral-GTP levels were determined using Ras and Ral Activation Assay Kits (Millipore) respectively, following the manufacturer's instructions.

### Cell culture, viral transduction, and siRNA transfection

All human melanoma cell lines including WM983C (BRAF^V600E^), UACC257 (BRAF^V600E^), IGR1 (BRAF^V600K^), and WM88 (BRAF^V600E^) were maintained in RPMI1640 Medium (HyClone) supplemented with 10% FBS (HyClone) and 1% Penicillin/streptomycin (Invitrogen) at 37°C with 5% CO_2_. All the cell lines were authenticated using short tandem repeat (STR) DNA profiling by Bio-Synthesis, Inc. Transfection of plasmids and siRNAs was performed using Lipofectamine 2000 (Invitrogen). Viral packaging plasmids along with shRNA constructs or cDNA constructs (delta 8.2 and VSVG for pLKO.1 vectors and pHit60 and MP2-VSVG for pQCXP-puro-gateway vectors) were transfected into 293T cells. Transduced cells with viral supernatant were selected in 1–2 μg/ml puromycin for 2~3 days. In addition, melanoma cells were transduced with recombinant adenovirus encoding RASA1 or GFP (Vector biolabs) and were collected at 72 hr post-infection for protein analysis.

### Soft agar-colony formation assays

Soft agar-colony formation assays were performed on 6-well plates in triplicate at a density of 10,000 cells/well in medium containing 0.35% agarose layered over 0.5% agarose. Each well was allowed to solidify and subsequently covered with 1 ml culture media, which was refreshed every 3 days. After growing for indicated days, colonies were stained with 0.05% iodonitrotetrazolium chloride (Sigma), followed by counting colonies in 3 representative fields per well. All the statistical analysis was done by two-tailed *t-test*. Each experiment was repeated at least two times in triplicates and representative results from one experiment are shown.

### Mouse xenograft assay

Two million IGR1 cells with pLKO.1 vector or with shRASA1c (*n* = 8 each) or three million WM 983C cells with control, RASA1^wt^, or RASA1^Y472H^ were inoculated subcutaneously (*n* = 7 each) to seven week-old female nude mice (Crl:NU-Foxn1 ^Nu/Nu^). On day 48 for IGR1 and day 29 for WM983C post implantation, mice were euthanatized and tumors were excised for weight measurement and analysis. The experiment was repeated twice and similar effects were observed. Data from one experiment is shown. All animals were maintained according to the guidelines of the Comparative Medicine of the University of South Florida.

### Immunohistochemistry

Construction of a melanoma TMA containing nevi and primary and metastatic melanoma samples was described previously [37]. Following a heat-induced epitope retrieval step (0.01 M citrate buffer, pH 6.0), a TMA section was incubated with RASA1 antibody, followed by biotinylated secondary antibody (Vector labs) and Vectastain ABC-AP (alkaline phosphatase) system (Vector labs). All sections were visualized with Vulcan Fast Red Chromogen kit (Biocarta) and counterstained with hematoxylin. Stained slides were scanned and scored by a dermatopathologist (JLM) as absent or present. Cores without enough tissue were not scored. *P-*values were calculated using Fisher's exact test comparing each group to Clark's nevi group and 95% confidence intervals (CI) are exact binomial estimates.

### Retrospective melanoma cohort

Genetic, genomic, clinical, and expression data for archived metastatic melanoma samples were obtained from the Total Cancer Care (TCC) database, which is described in Supplementary Materials and Methods. Kaplan-Meier survival curves were estimated for both high and low RASA1 expressed groups. Cox regression model based on robust variance estimates is used to infer hazard ratio (HR) of RASA1 expression level for death while adjusting the effect of other covariates such as age and gender, and taking into account the correlation between tumor samples from the same patient. Data were analyzed using open-source statistical software R 3.1.0 (http://www.R-project.org/). This study received expedited approval by the Institutional Review Board at the University of South Florida: Category 5 (45CFR46.110 and 21 CFR 56.110).

## SUPPLEMENTARY MATERIALS FIGURES AND TABLES


