# Supplementary material for: Inactivation of RASA1 promotes melanoma tumorigenesis via R-Ras activation
Source: Oncotarget. 2016 Mar 16;7(17):23885–96. doi: 10.18632/oncotarget.8127 (PMC5029671; doi:10.18632/oncotarget.8127)
Supplement: Supplementary file 1 [file oncotarget-07-23885-s001.pdf]

Inactivation of RASA1 promotes melanoma tumorigenesis via R-Ras activation

Supplementary Materials

Proximity analysis

Clustered mutations within specific protein regions across multiple individuals were identified as described in (21) by querying 1) the number of mutations on the same transcript within the proximity limit (10 amino acids) and 2) the distance between the closest mutations. The resulting mutational clusters were sorted and annotated to identify recurrent mutations in genes with particular relevance to metastatic melanoma.

Protein analysis

Cells or homogenized tissues were lysed in RIPA buffer containing protease inhibitors (Roche) and phosphatase inhibitors (Calbiochem). Proteins were fractionated on 10% SDS-polyacrylamide gel or 4–12% Novex Bis-Tris gel (Invitrogen) electrophoresis and then transferred to PVDF membrane (EMD Millipore).

DNA constructs, viral vectors, shRNAs, and siRNAs

The human *RASA1* complementary DNA (cDNA) was purchased from Open Biosystems (BC033015, clone ID# 4829173) and RASA1 Y472H and L481F mutation were introduced with QuickChange XL Site-Directed Mutagenesis Kit (Stratagene) according to the manufacturer's instructions. Coding regions of each wild type and mutants were cloned into the pQCXP-puro- (a gift of Dr. Scott and Dr. Chin) via Gateway cloning system (Invitrogen). pCMV6-XL4-RASA1 was purchased from OriGene. The lentiviral shRNAs in pLKO.1 vector, shRASA1b (5′-CCTGACATCAATAGATTTGAA-3′), shRASA1c (5′-CCCTACATGGAAGGTGTCAAT-3′) were from Open Biosystems. All constructs were sequence verified. The siRNAs targeting *RASA1* (SASI-Hs01-00220764 (#764), -00220766 (#766), -00220768 (#768)) were from Sigma-Aldrich, BLOCK-iT (si-Ctrl) from Invitrogen, siR-Ras (A: 5′-CTCGGCCAAACTGCGTCTCAA-3′; B: 5′-CCGGAA ATACCAGGAACAAGA-3′) and AllStars negative control (siCtrl) from Qiagen. siRNA targeting Ral-A (ON-TARGET plus Human RALA(5895) siRNA-SMART pool) was obtained from Dharmacon. Recombinant adenovirus encoding RASA1 or GFP was purchased from Vector Biolabs.

Retrospective melanoma cohort study

Genetic, genomic, clinical, and expression data for archived metastatic melanoma samples were obtained from the Total Cancer Care (TCC) database. TCC analyzed metastatic melanomas from patients treated at the Moffitt Cancer Center for mRNA expression on Affymetrix HuRSTA-2a520709 GeneChips (Affymetrix, Santa Clara, CA) under Liberty IRB#12.11.0023. TCC also performed targeted exome sequencing for 1,403 genes. Age of onset, gender, location of tumor, histological features, and overall survival as well as mutation status and expression level of *RASA1* and *BRAF* mutation status were obtained. Since two probe sets targeted the overlapping regions in exon 24 of *RASA1* and showed highly significant positive correlation (r = 0.964, Supplementary Figure S2A), average intensity of the two *RASA1* probe sets was utilized. Overall survival was determined as the length of time (months) from the date of diagnosis for that primary tumor until death or last follow up.

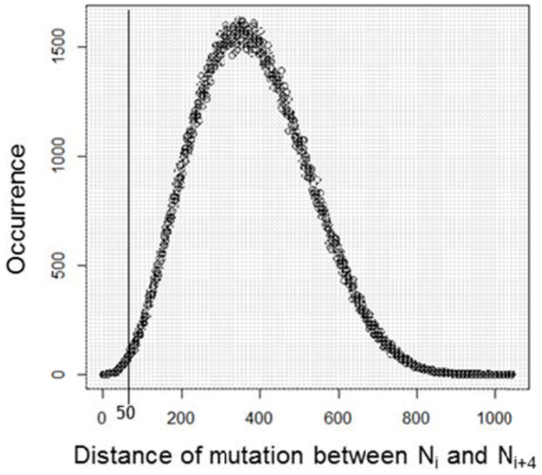

Supplementary Figure S1: Significant clustering of known *RASA1* mutations in melanoma. Probability of observing any 5 mutations within a window of 50 amino acids in any location (P) is 0.000965 if 10 mutations are randomly distributed on a protein of 1047 amino acids length. (method) Among 1 to 1047, 10 numbers were randomly selected allowing repeat. These numbers were sorted from smallest to biggest and then distance between Ni and Ni+4 were calculated. Following 10,000 repeats, the data was plotted to show occurrence for each distance between Ni and Ni+4 mutations. Probability was calculated by dividing total number of occurrences that 5 mutations were localized within 50 amino acids window with the total number of any observed occurrences.

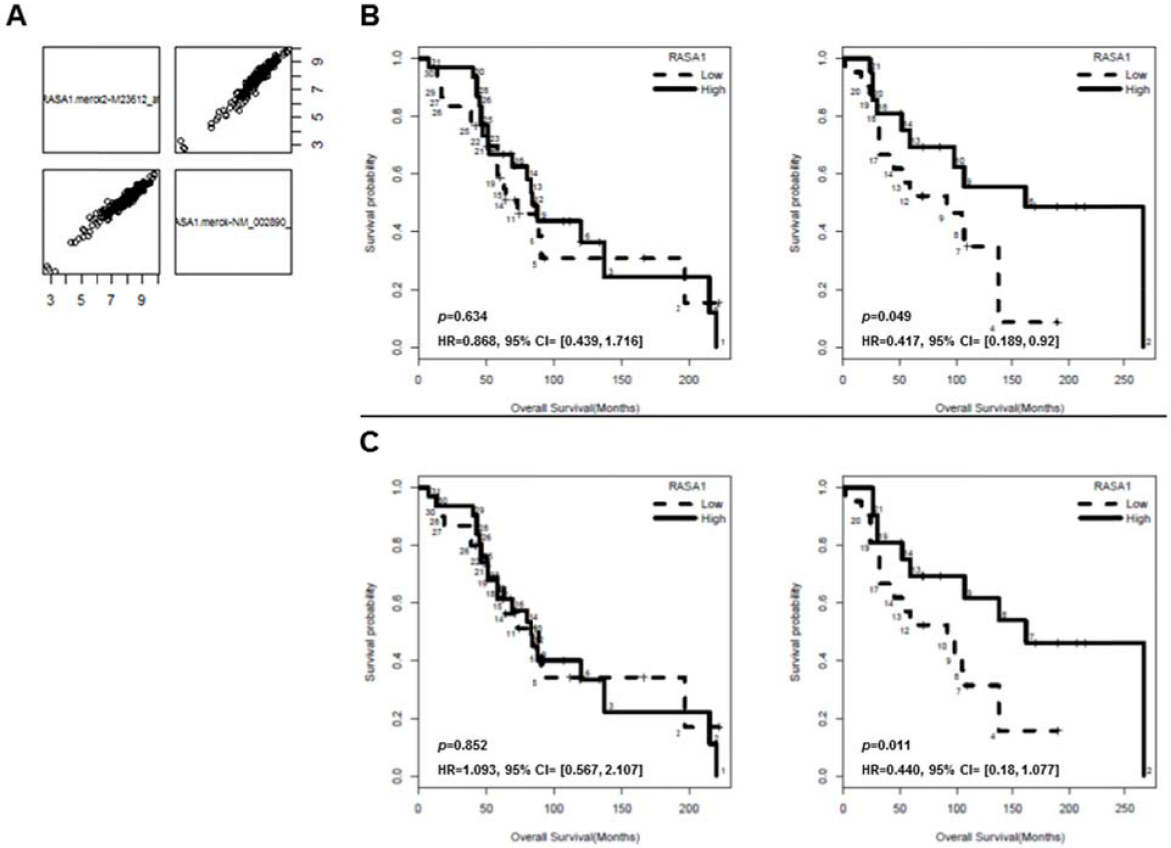

Supplementary Figure S2: Association of *RASA1* mRNA level with overall survival of melanoma patients. (**A**) Correlation of merck2-M23612_at and merck-NM_002890_at probe sets for *RASA1* mRNA level in each sample (r = 0.964). (**B** and **C**) Kaplan-Meier overall survival curves for *BRAF* wild type (left) and mutant (right) groups for the probe set merck2-M23612_at (B) and merck-NM_002890_at (C). Samples were classified based on *RASA1* mRNA level as high (above median) and low (below median).

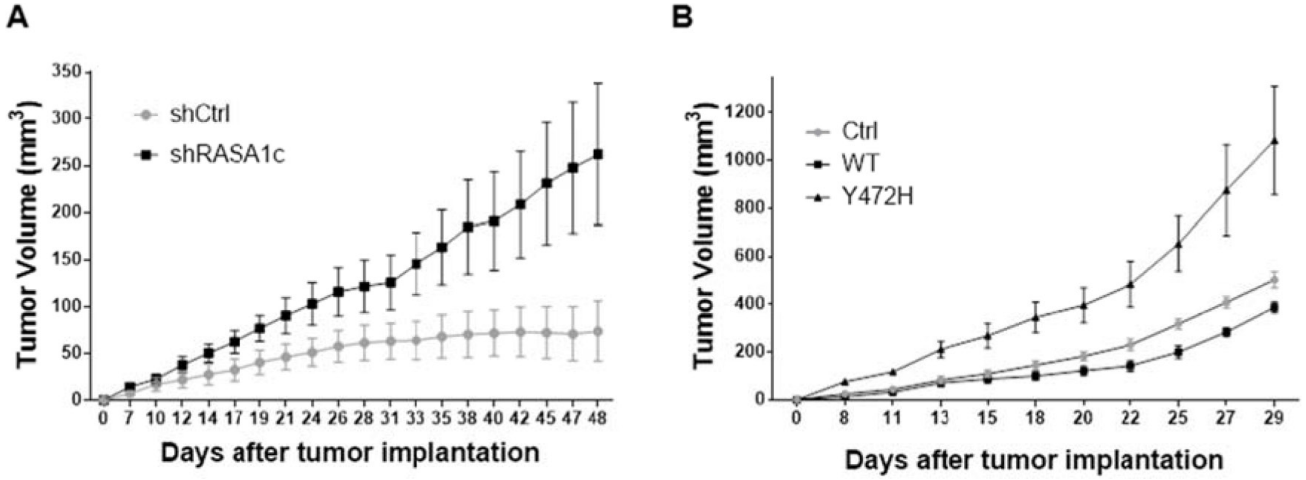

Supplementary Figure S3: RASA1 inhibits growth of IGR1 and WM983C subcutaneous tumors. Mean volume of the subcutaneous tumors with SEM is shown over time post injection. (**A**) shows data from IGR1 cells with shRASA1c or vector control (shCtrl) (n = 8 each) and (**B)** is for WM983C ($BRAF^{V600E}$) cells with vector control (Ctrl), wild type (WT), or Y472H mutant RASA1 (n = 7 each). Tumor size was measured every other day with calipers, and tumor volumes were calculated using the formula [Tumor volume (mm^3) = $(width)^2 \times (length) /2$]. Repeated ANOVA approach was used to test the time (within-subject factor) and the interaction between group and time (between-subject factor) on tumor volume. The p-value for the time and interaction is 0.002 and 0.047, respectively. When Tukey method to control Type I error inflation was applied to adjust for the multiple comparison, the difference between shCtrl and shRASA1c groups become statistically significant from day 42 (p = 0.048), day 45 (p = 0.042), day 47 (p = 0.035) and up to day 48 (p = 0.038). Tumor volumes were log transformed for the statistical analysis. When Tukey-Kramer method was applied, difference between control (Ctrl) and Y472H group is significant from day 8 (adjusted $p < 0.001$). Difference of tumor volume between control (Ctrl) and wild type (WT) group is marginally significant on day 15 (adjusted p = 0.055).

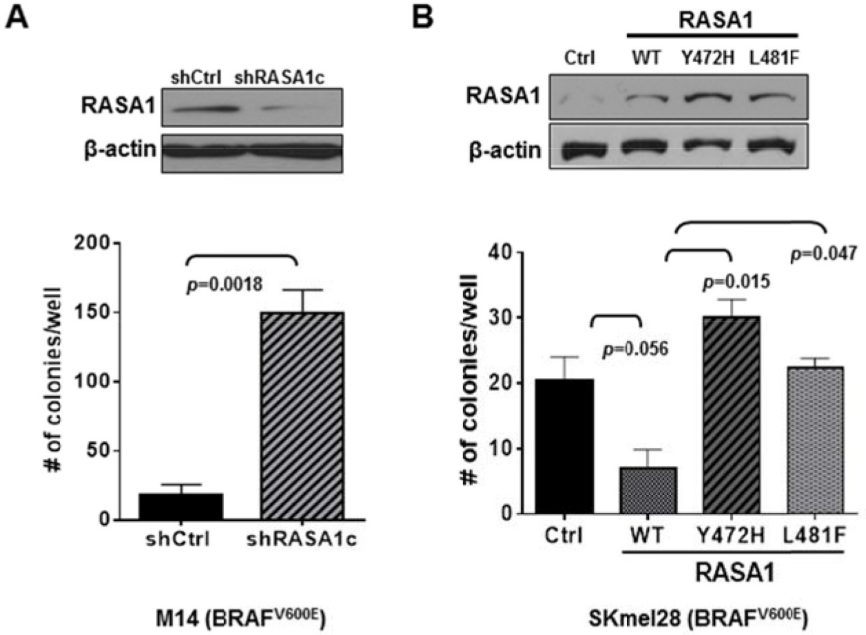

Supplementary Figure S4: RASA1 suppresses anchorage-independent growth *in vitro*. Reduced RASA1 expression by shRASA1c in M14 promotes anchorage-independent colony formation compared to control cell with vector control (shCtrl) (**A**). Conversely, expression of RASA1 wild type (WT), but not Y472H and L481F mutants, suppresses colony growth on soft agar *in vitro* in SKmel28 ($BRAF^{V600E}$) (**B**) cells when transduced with retroviruses generated with pQCXP viral vector (B). Graph shows the mean number of colonies grown on soft agar on day 17 (A) or day 21 (B) per well with SD.

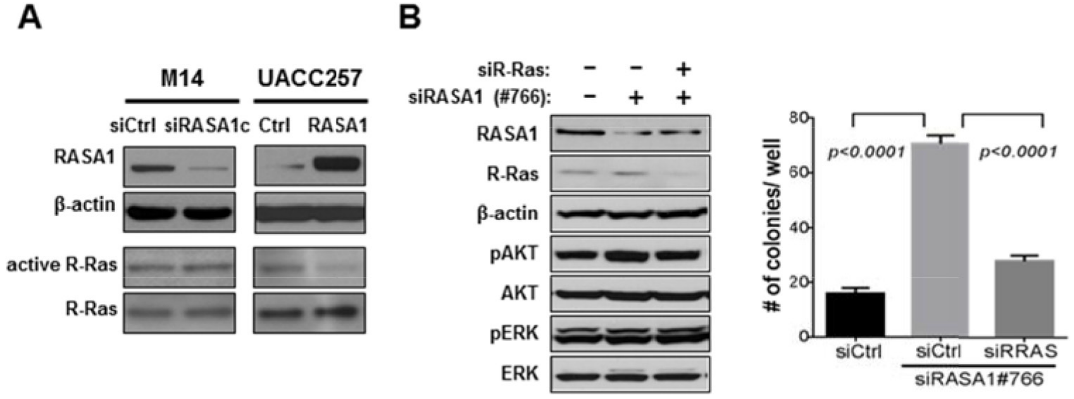

Supplementary Figure S5: RASA1 knockdown promotes R-Ras activation, which regulates growth in soft agar. The effect of RASA1 modulation on R-Ras activation is addressed in M14 and UACC257 cells (**A**). Levels of RASA1, β-actin, and R-Ras were addressed by immunoblotting with indicated antibodies. Activities of R-Ras were analyzed by pulling-down GTP-bound active R-Ras. (**B**) Reduced RASA1 expression with #766 siRNA increased soft agar colony formation, which was reversed by reduced R-Ras expression with *RRAS* siRNA in IGR1 cells. Number of soft-agar colonies per well on day 14 counted under the microscope are shown as mean +/– SD.

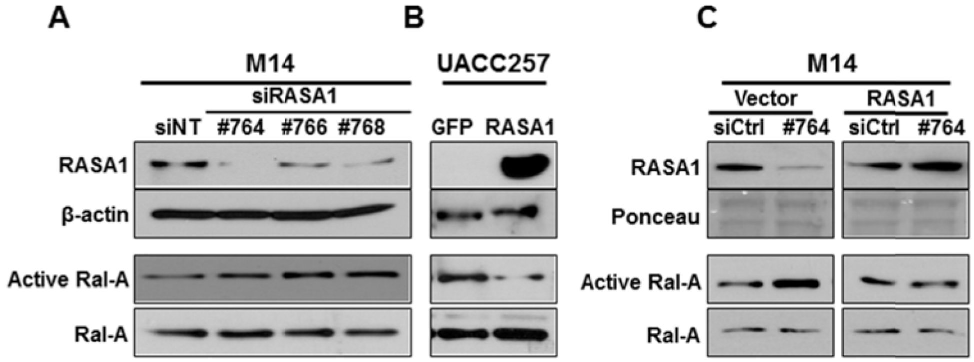

Supplementary Figure S6: RASA1 regulates R-Ras activation in M14 and UACC257 cells. The effect of RASA1 modulation on major effectors of Ras signaling is addressed in M14 and UACC257 cells. Transfection of siRNAs (#764, 766, or 768) targeting RASA1 in M14 cells (**A** and **C**) reduced RASA1 expression and increased GTP-bound active Ral-A compared to control cells with siNT. RASA1 overexpression by transduction with recombinant adenovirus encoding RASA1 suppressed Ral-A activation in UACC257 cells compared to control transduced with adenovirus encoding GFP (**B**).Co-transfection of pCMV-XL4-RASA1 with #764 siRNA rescued RASA1 expression and suppressed Ral-A activation (C).

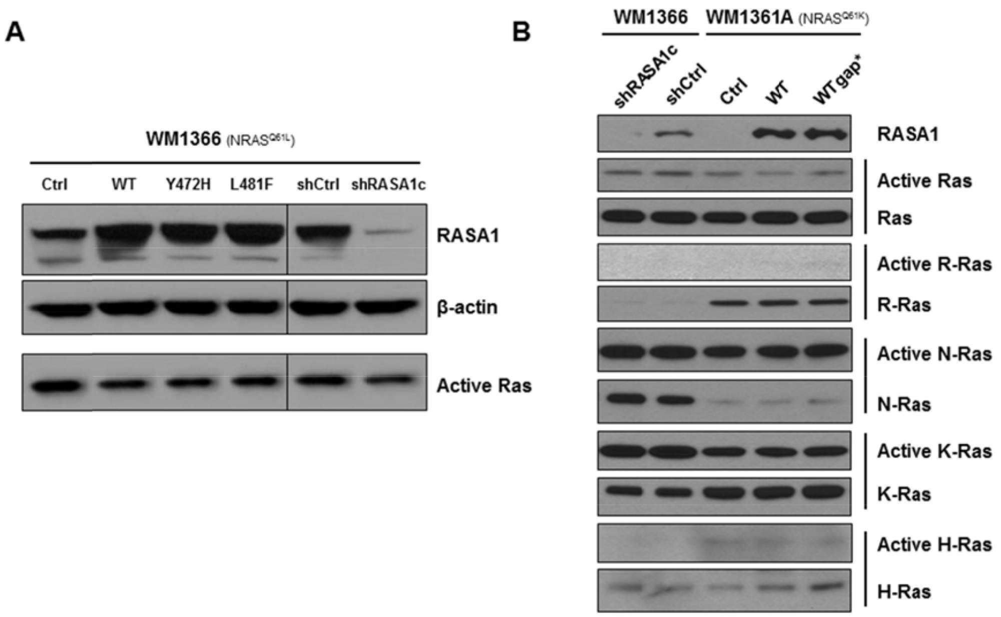

Supplementary Figure S7: Effect of RASA1 on Ras activity in *NRAS* mutant melanoma cell lines. Active GTP-bound Ras in human melanoma cell lines with activating *NRAS* mutations, WM1366 (NRAS^{Q61L}) and WM1361A (NRAS^{Q61K}) with RASA1 over-expression or knock-down, is probed with isoform specific antibodies against H-, K-, N-, and R-Ras or with pan-Ras antibody.

Supplementary Table S1: List of proximal mutations

Mutations Within Proximity	Nearest Mutation	Gene	Transcript	Affected Amino Acid (s)	Chr	Start	Stop	Ref Allele	Var Allele	Sample
9	0	BRAF	NM_004333.4	600	7	140099605	140099605	A	T	H_LX-WU_ MEL_521
9	0	BRAF	NM_004333.4	600	7	140099606	140099606	C	T	H_LX-80
9	0	BRAF	NM_004333.4	600	7	140099605	140099605	A	T	H_LX-80
9	0	BRAF	NM_004333.4	600	7	140099605	140099605	A	T	H_LX-174
9	0	BRAF	NM_004333.4	600	7	140099606	140099606	C	T	H_LX-WU_ MEL_521
9	0	BRAF	NM_004333.4	600	7	140099605	140099605	A	T	H_LX-123
9	0	BRAF	NM_004333.4	600	7	140099605	140099605	A	T	H_LX-WU_ Mel_218
9	0	BRAF	NM_004333.4	600	7	140099605	140099605	A	T	H_LX-WU_ MEL_522
9	0	BRAF	NM_004333.4	600	7	140099605	140099605	A	T	H_LX-139
9	0	BRAF	NM_004333.4	600	7	140099605	140099605	A	T	H_LX-WU_ MEL_500
6	0	BRAF	NM_004333	600	7	140099605	140099605	A	T	MEL2
6	0	BRAF	NM_004333	600	7	140099605	140099606	AC	TT	MEL5
6	0	BRAF	NM_004333	600	7	140099605	140099605	A	T	MEL13
6	0	BRAF	NM_004333	600	7	140099605	140099605	A	T	MEL10
6	0	BRAF	NM_004333	600	7	140099605	140099605	A	T	MEL12
6	0	BRAF	NM_004333	600	7	140099605	140099605	A	T	MEL7
6	0	BRAF	NM_004333	600	7	140099605	140099605	A	T	MEL3
6	0	MUC4	ENST00000405167	2317	3	196995895	196995895	G	C	H_LX-167
6	0	MUC4	ENST00000405167	2317	3	196995895	196995895	G	C	H_LX-172
6	0	MUC4	ENST00000405167	2317	3	196995895	196995895	G	C	H_LX-80
5	0	MUC4	ENST00000405167	2309	3	196995920	196995920	T	C	H_LX-123
5	0	MUC4	ENST00000405167	2309	3	196995920	196995920	T	C	H_LX-WU_ MEL_503
5	0	MUC4	ENST00000405167	2309	3	196995920	196995920	T	C	H_LX-159
3	0	ENSG00000215548	ENST00000358464	68	20	28227957	28227958	0	AGA	H_LX-174
3	0	ENSG00000215548	ENST00000358464	68	20	28227957	28227958	0	AGA	H_LX-139
3	0	ENSG00000215548	ENST00000358464	68	20	28227957	28227958	0	AGA	H_LX-WU_ MEL_503
3	5	ENSG00000215548	ENST00000358464	63	20	28227943	28227943	T	G	H_LX-159
3	3	MUC4	ENST00000405167	2320	3	196995888	196995888	G	A	H_LX-159
2	0	ENSG00000211726	ENST00000390373	55	7	141856587	141856587	C	T	MEL6
2	3	ENSG00000211726	ENST00000390373	52	7	141856579	141856579	A	G	MEL9
2	0	ENSG00000211726	ENST00000390373	55	7	141856587	141856587	C	T	MEL5
2	0	HNF4G	NM_004133	78	8	76618744	76618744	C	T	MEL6
2	0	HNF4G	NM_004133	78	8	76618744	76618744	C	T	MEL5
2	0	HNF4G	NM_004133	78	8	76618744	76618744	C	T	MEL9
2	3	LOC100134712	XM_001714845	241	7	10441870	10441870	C	T	MEL12
2	4	LOC100134712	XM_001714845	245	7	10441858	10441858	T	C	MEL9
2	3	LOC100134712	XM_001714845	238	7	10441878	10441878	C	A	MEL7
2	0	NRAS	NM_002524	61	1	115058052	115058052	T	C	MEL9
2	0	NRAS	NM_002524	61	1	115058053	115058053	G	T	MEL8
2	0	NRAS	NM_002524	61	1	115058052	115058052	T	C	MEL1
2	2	PDZD2	NM_178140.2	24	5	31835183	31835184	0	G	H_LX-80

2	2	PDZD2	NM_178140.2	22	5	31835177	31835178	0	C	H_LX-80
2	2	PDZD2	NM_178140.2	26	5	31835188	31835189	0	A	H_LX-80
2	1	PXDNL	NM_144651	469	8	52522236	52522236	G	A	MEL4
2	1	PXDNL	NM_144651	468	8	52522240	52522240	G	A	MEL12
2	5	PXDNL	NM_144651	463	8	52522255	52522255	C	T	MEL7
1	0	ABCA13	NM_152701.2	4730	7	48538486	48538486	G	A	H_LX-WU_ MEL_522
1	0	ABCA13	NM_152701.2	4730	7	48538485	48538485	G	A	H_LX-WU_ MEL_522
1	10	ABCA13	NM_152701	4594	7	48530328	48530328	C	T	MEL9
1	10	ABCA13	NM_152701	4604	7	48530359	48530359	C	T	MEL11
1	0	ACSS3	NM_024560	634	12	80171485	80171485	C	T	MEL3
1	0	ACSS3	NM_024560	634	12	80171485	80171485	C	T	MEL4
1	4	ADAM7	NM_003817	567	8	24406490	24406490	C	T	MEL6
1	4	ADAM7	NM_003817	563	8	24406477	24406477	G	A	MEL11
1	2	ADAMTS20	NM_025003	985	12	42112516	42112516	C	T	MEL5
1	2	ADAMTS20	NM_025003	987	12	42112510	42112510	C	T	MEL2
1	8	ADAMTSL3	NM_207517	460	15	82352557	82352557	G	A	MEL9
1	8	ADAMTSL3	NM_207517	452	15	82352532	82352532	G	A	MEL11
1	0	AHCTF1	NM_015446.3	87	1	245146182	245146182	G	A	H_LX-159
1	0	AHCTF1	NM_015446.3	87	1	245146182	245146182	G	A	H_LX-WU_ MEL_21
1	3	ANK3	NM_020987	919	10	61564120	61564120	G	A	MEL6
1	9	ANK3	NM_020987	1632	10	61505750	61505750	G	A	MEL5
1	3	ANK3	NM_020987	916	10	61564129	61564129	G	A	MEL8
1	9	ANK3	NM_020987	1623	10	61505777	61505777	C	T	MEL1
1	2	ANKRD32	NM_032290	82	5	94049998	94049998	C	0	MEL12
1	2	ANKRD32	NM_032290	80	5	94049992	94049992	G	A	MEL9
1	1	ASCL5	XM_001719321	81	1	199351597	199351597	C	T	MEL10
1	1	ASCL5	XM_001719321	82	1	199351595	199351595	G	A	MEL9
1	3	ASXL3	NM_030632	533	18	29572964	29572964	T	G	MEL6
1	3	ASXL3	NM_030632	536	18	29572974	29572974	G	T	MEL4
1	8	BARX2	NM_003658	92	11	128811943	128811943	G	A	MEL9
1	8	BARX2	NM_003658	84	11	128811919	128811919	C	T	MEL5
1	10	BLMH	NM_000386	387	17	25618085	25618085	C	T	MEL9
1	10	BLMH	NM_000386	397	17	25618055	25618055	G	T	MEL10
1	0	BRAF	NM_004333	574	7	140100477	140100477	G	A	MEL4
1	0	BRAF	NM_004333	574	7	140100476	140100476	T	A	MEL9
1	10	BTN2A3	ENST00000244504	197	6	26531649	26531649	C	T	MEL6
1	10	BTN2A3	ENST00000244504	187	6	26531621	26531621	G	A	MEL12
1	7	C4orf18	NM_001031700	154	4	159311518	159311518	C	T	MEL9
1	7	C4orf18	NM_001031700	161	4	159311497	159311497	C	T	MEL6
1	9	C4orf37	NM_174952	358	4	98981079	98981079	C	T	MEL9
1	9	C4orf37	NM_174952	349	4	98981107	98981107	C	T	MEL8
1	2	C6orf190	NM_001010923	505	6	128175966	128175966	G	A	MEL12
1	2	C6orf190	NM_001010923	503	6	128175972	128175972	C	T	MEL3
1	7	C6orf222	NM_001010903	485	6	36399065	36399065	G	A	MEL11
1	7	C6orf222	NM_001010903	492	6	36398195	36398195	G	A	MEL12
1	7	CA2	NM_000067	145	8	86573887	86573887	T	0	MEL9
1	7	CA2	NM_000067	138	8	86573865	86573865	G	A	MEL7

1	0	CASP8	NM_001080125	107	2	201839596	201839596	C	T	MEL10
1	0	CASP8	NM_001080125	107	2	201839596	201839596	C	T	MEL11
1	8	CCDC54	NM_032600	277	3	108579954	108579954	C	T	MEL9
1	8	CCDC54	NM_032600	285	3	108579977	108579977	G	A	MEL3
1	0	CCL7	ENST00000394627	71	17	29622330	29622330	C	T	MEL6
1	0	CCL7	ENST00000394627	71	17	29622330	29622330	C	T	MEL4
1	9	CDH10	NM_006727	656	5	24523929	24523929	C	T	MEL9
1	9	CDH10	NM_006727	665	5	24523903	24523903	C	T	MEL10
1	10	CDK2	NM_001798	204	12	54651116	54651116	C	T	MEL9
1	10	CDK2	NM_001798	214	12	54651147	54651147	G	A	MEL9
1	1	CEP350	NM_014810	125	1	178227960	178227960	G	A	MEL9
1	1	CEP350	NM_014810	124	1	178227957	178227957	C	T	MEL4
1	0	CER1	NM_005454	30	9	14712583	14712583	G	A	MEL6
1	0	CER1	NM_005454	30	9	14712583	14712583	G	A	MEL13
1	0	CNKSR3	NM_173515.2	457	6	154773169	154773169	C	T	H_LX-WU_ MEL_21
1	0	CNKSR3	NM_173515.2	457	6	154773168	154773168	C	T	H_LX-WU_ MEL_21
1	5	COL2A1	NM_001844	624	12	46664612	46664612	C	T	MEL9
1	5	COL2A1	NM_001844	629	12	46664595	46664595	C	T	MEL9
1	3	COL9A1	NM_001851.4	520	6	71021627	71021627	C	T	H_LX-139
1	3	COL9A1	NM_001851.4	517	6	71021768	71021768	C	T	H_LX-139
1	6	CRISP2	NM_003296	193	6	49771534	49771534	G	A	MEL11
1	6	CRISP2	NM_003296	199	6	49771516	49771516	C	T	MEL9
1	0	CSMD1	ENST00000335551	162	8	3252876	3252876	G	A	H_LX-WU_ MEL_522
1	0	CSMD1	ENST00000335551	162	8	3252876	3252876	G	A	MEL10
1	0	CSMD1	NM_033225	672	8	3252888	3252888	G	A	MEL6
1	0	CSMD1	NM_033225	672	8	3252888	3252888	G	A	MEL3
1	10	CSMD1	NM_033225.4	838	8	3241208	3241208	G	A	H_LX-WU_ MEL_500
1	10	CSMD1	NM_033225.4	828	8	3241237	3241237	C	T	H_LX-80
1	2	CYFIP2	ENST00000398252	192	5	156718638	156718638	C	T	MEL6
1	2	CYFIP2	ENST00000398252	190	5	156718632	156718632	G	A	MEL8
1	0	CYP4Z2P	ENST00000294342	47	1	47138555	47138555	G	A	MEL6
1	0	CYP4Z2P	ENST00000294342	47	1	47138555	47138555	G	A	MEL12
1	0	CYTH4	NM_013385	285	22	36037019	36037019	G	A	MEL6
1	0	CYTH4	NM_013385	285	22	36037019	36037019	G	A	MEL7
1	2	DBC1	NM_014618	688	9	120969406	120969406	G	A	MEL3
1	2	DBC1	NM_014618	690	9	120969400	120969400	G	A	MEL1
1	8	DCDC2	NM_016356	377	6	24286734	24286734	C	T	MEL6
1	8	DCDC2	NM_016356	369	6	24286756	24286756	C	T	MEL9
1	2	DEFA6	NM_001926	84	8	6769802	6769802	G	A	MEL6
1	2	DEFA6	NM_001926	86	8	6769796	6769796	C	T	MEL6
1	6	DISC1	NM_018662	563	1	229997663	229997663	A	G	MEL9
1	6	DISC1	NM_018662	557	1	229997646	229997646	T	C	MEL9
1	4	DKFZp779B1634	XM_292717	725	18	12116035	12116035	G	A	MEL6
1	4	DKFZp779B1634	XM_292717	729	18	12116048	12116048	G	A	MEL4
1	0	DNAH11	ENST00000328843	940	7	21606081	21606081	C	T	H_LX-WU_ MEL_522

1	0	DNAH11	ENST00000328843	940	7	21606080	21606080	C	T	H_LX-WU_ MEL_522
1	10	DNAH8	NM_001371	2810	6	38975546	38975546	G	A	MEL11
1	10	DNAH8	NM_001371	2820	6	38975576	38975576	G	A	MEL12
1	5	DNAH9	NM_001372	1741	17	11548314	11548314	G	A	MEL5
1	5	DNAH9	NM_001372	1746	17	11548331	11548331	G	A	MEL11
1	6	DSCAM	NM_001389	224	21	40647525	40647525	G	C	MEL11
1	6	DSCAM	NM_001389	230	21	40647508	40647508	C	T	MEL12
1	10	DSG3	NM_001944	138	18	27293033	27293033	G	A	MEL9
1	10	DSG3	NM_001944	128	18	27293004	27293004	G	A	MEL6
1	5	DSP	NM_004415	1312	6	7525357	7525357	C	T	MEL5
1	5	DSP	NM_004415	1307	6	7525342	7525342	C	T	MEL9
1	3	EGFLAM	NM_152403	157	5	38388114	38388114	G	A	MEL5
1	3	EGFLAM	NM_152403	154	5	38388106	38388106	C	T	MEL8
1	0	ENSG00000205693	ENST00000381273	136	12	27807537	27807537	A	0	MEL10
1	0	ENSG00000205693	ENST00000381273	136	12	27807535	27807535	T	0	MEL10
1	6	ENSG00000211744	ENST00000390391	8	7	141990254	141990254	G	A	MEL9
1	6	ENSG00000211744	ENST00000390391	14	7	141990271	141990271	T	G	MEL5
1	4	ENSG00000212884	ENST00000391571	1	NT_113930	117370	117370	A	G	H_LX-WU_ MEL_522
1	4	ENSG00000212884	ENST00000391571	5	NT_113930	117384	117384	C	A	H_LX-139
1	8	ENSG00000214481	ENST00000398401	349	6	78689663	78689663	C	T	MEL3
1	8	ENSG00000214481	ENST00000398401	357	6	78689689	78689689	C	A	MEL4
1	9	ENSG00000215231	ENST00000399853	123	5	5110909	5110909	G	A	MEL4
1	9	ENSG00000215231	ENST00000399853	114	5	5110882	5110882	G	A	MEL6
1	7	EPB41L1	ENST00000344237	679	20	34258725	34258725	C	T	MEL4
1	7	EPB41L1	ENST00000344237	672	20	34258704	34258704	G	A	MEL9
1	1	EPHA3	NM_005233.5	317	3	89472890	89472890	C	T	H_LX-167
1	1	EPHA3	NM_005233.5	316	3	89472887	89472887	G	A	H_LX-123
1	2	EPHA7	NM_004440	223	6	94177105	94177105	C	T	MEL6
1	2	EPHA7	NM_004440	225	6	94177098	94177098	G	A	MEL5
1	0	F5	NM_000130.4	1321	1	167776991	167776991	T	G	H_LX-139
1	0	F5	NM_000130.4	1321	1	167776991	167776991	T	G	H_LX-123
1	0	FAM133A	NM_173698	36	X	92851180	92851180	C	T	MEL6
1	0	FAM133A	NM_173698	36	X	92851181	92851181	G	A	MEL7
1	0	FAM19A3	NM_001004440	152	1	113068278	113068278	C	T	MEL4
1	0	FAM19A3	NM_001004440	152	1	113068279	113068279	C	T	MEL4
1	0	FLG	NM_002016	888	1	150551323	150551323	C	T	MEL6
1	0	FLG	NM_002016	888	1	150551323	150551323	C	T	MEL5
1	0	FMN2	NM_020066.4	197	1	238322192	238322194	GGC	0	H_LX-172
1	0	FMN2	NM_020066.4	197	1	238322192	238322194	GGC	0	H_LX-139
1	2	FNBP1	NM_015033	309	9	131727122	131727122	G	A	MEL3
1	2	FNBP1	NM_015033	307	9	131727128	131727128	C	T	MEL9
1	4	FREM3	XM_094074	1025	4	144838206	144838206	C	T	MEL6
1	4	FREM3	XM_094074	1029	4	144838194	144838194	G	A	MEL12
1	1	FRG1	NM_004477.2	145146	4	191115549	191115550	0	A	H_LX-159
1	1	FRG1	NM_004477.2	144	4	191113301	191113301	G	C	H_LX-WU_ Mel_218
1	0	FRG1	NM_004477.2	172173	4	191115630	191115632	AGG	0	H_LX-WU_ MEL_522

1	0	FRG1	NM_004477.2	172173	4	191115630	191115632	AGG	0	H_LX-WU_ MEL_503
1	0	FRG1B	ENST00000278882	77	20	28241887	28241887	G	A	H_LX-80
1	0	FRG1B	ENST00000278882	77	20	28241887	28241887	G	A	H_LX-189
1	5	GABRA6	NM_000811	79	5	161048544	161048544	G	A	MEL6
1	5	GABRA6	NM_000811	74	5	161046582	161046582	G	A	MEL7
1	0	GARNL1	NM_194301.1	1149,1150	14	35211931	35211932	0	G	H_LX-139
1	0	GARNL1	NM_194301.1	1150	14	35211929	35211930	0	A	H_LX-139
1	0	GARNL1	NM_194301	1708	14	35166264	35166264	G	A	MEL9
1	0	GARNL1	NM_194301	1708	14	35166264	35166264	G	A	MEL6
1	3	GIMAP4	NM_018326	57	7	149900260	149900260	C	T	MEL4
1	3	GIMAP4	NM_018326	54	7	149900251	149900251	A	T	MEL13
1	8	GLT25D2	NM_015101	566	1	182174702	182174702	G	A	MEL9
1	8	GLT25D2	NM_015101	574	1	182174678	182174678	G	A	MEL9
1	8	GPR112	NM_153834	203	X	135233139	135233139	G	A	MEL6
1	8	GPR112	NM_153834	195	X	135233115	135233115	G	A	MEL7
1	5	GRIN2B	NM_000834	167	12	13798027	13798027	G	T	MEL9
1	5	GRIN2B	NM_000834	162	12	13798044	13798044	C	T	MEL7
1	0	GRIN2B	NM_000834.3	888	12	13608777	13608777	T	A	H_LX-167
1	0	GRIN2B	NM_000834.3	888	12	13608776	13608776	G	A	H_LX-167
1	0	GRIN3A	NM_133445.2	179	9	103539547	103539547	G	A	H_LX-167
1	0	GRIN3A	NM_133445.2	179	9	103539547	103539547	G	C	H_LX-WU_ Mel_218
1	7	HDAC9	NM_178425	194	7	18600093	18600093	C	T	MEL9
1	7	HDAC9	NM_178425	187	7	18600073	18600073	C	T	MEL9
1	9	HEATR5A	ENST00000389961	236	14	30933074	30933074	A	G	MEL11
1	9	HEATR5A	ENST00000389961	245	14	30933048	30933048	T	A	MEL4
1	4	HHLA2	NM_007072	306	3	109559611	109559611	G	A	MEL11
1	4	HHLA2	NM_007072	302	3	109559601	109559601	G	A	MEL9
1	7	HIPK1	NM_198268	110	1	114284857	114284857	G	T	MEL4
1	7	HIPK1	NM_198268	117	1	114284877	114284877	C	T	MEL6
1	4	HTR2C	ENST00000371950	164	X	114047442	114047442	G	A	H_LX-159
1	4	HTR2C	ENST00000371950	160	X	114047430	114047430	G	A	MEL6
1	0	IGF2R	NM_000876.2	1766	6	160417000	160417000	T	0	H_LX-WU_ MEL_522
1	0	IGF2R	NM_000876.2	1766	6	160416998	160416998	T	C	H_LX-WU_ MEL_522
1	7	ILDR1	NM_175924	207	3	123202871	123202871	G	A	MEL7
1	7	ILDR1	NM_175924	200	3	123202893	123202893	A	G	MEL3
1	6	KCND2	NM_012281	178	7	119702454	119702454	C	T	MEL7
1	6	KCND2	NM_012281	184	7	119702472	119702472	G	A	MEL12
1	4	KCNJ15	NM_002243	238	21	38593765	38593765	C	T	MEL3
1	4	KCNJ15	NM_002243	242	21	38593778	38593778	C	T	MEL4
1	10	KIAA0564	NM_015058	1538	13	41087221	41087221	C	T	MEL9
1	10	KIAA0564	NM_015058	1548	13	41087190	41087190	C	T	MEL5
1	3	KIAA1109	NM_015312	4362	4	123488340	123488340	C	T	MEL5
1	3	KIAA1109	NM_015312	4359	4	123488331	123488331	C	T	MEL6
1	7	LBR	NM_002296	97	1	223676478	223676478	G	A	MEL9
1	7	LBR	NM_002296	104	1	223676457	223676457	G	A	MEL6
1	0	LCT	NM_002299.2	1081	2	136283145	136283145	G	A	H_LX-WU_ MEL_21

1	0	LCT	NM_002299.2	1081	2	136283146	136283146	G	A	H_LX-WU_MEL_21
1	0	LILRA5	NM_181879	264	19	59514417	59514417	G	A	MEL9
1	0	LILRA5	NM_181879	264	19	59514417	59514417	G	A	MEL11
1	4	LOC100129126	XM_001715328	170	7	63061752	63061752	G	A	MEL5
1	4	LOC100129126	XM_001715328	174	7	63061741	63061741	G	A	MEL9
1	4	LOC100129870	XM_001715927	167	5	79400173	79400173	C	T	MEL9
1	4	LOC100129870	XM_001715927	171	5	79400162	79400162	G	A	MEL9
1	7	LOC100131190	XM_001722162	124	21	41657798	41657798	C	T	MEL9
1	7	LOC100131190	XM_001722162	117	21	41657777	41657777	G	A	MEL9
1	6	LOC100131397	XM_001716553	122	5	16233496	16233496	C	T	MEL11
1	6	LOC100131397	XM_001716553	128	5	16233514	16233514	G	A	MEL4
1	2	LOC100134205	XM_001716692	183	6	4396100	4396100	G	A	MEL7
1	2	LOC100134205	XM_001716692	185	6	4396095	4396095	G	A	MEL9
1	9	LOC100134607	XM_001719062	70	7	55205928	55205928	G	A	MEL13
1	9	LOC100134607	XM_001719062	79	7	55205900	55205900	C	T	MEL5
1	8	LOC339760	XM_295058	381	2	127373586	127373586	G	A	MEL5
1	8	LOC339760	XM_295058	373	2	127373332	127373332	G	A	MEL7
1	1	LOC728667	XM_001128080	29	14	23367151	23367151	A	G	MEL12
1	1	LOC728667	XM_001128080	28	14	23367153	23367153	C	T	MEL4
1	5	LOC728944	XM_001128859	234	2	50667	50667	C	T	MEL6
1	5	LOC728944	XM_001128859	239	2	50652	50652	C	T	MEL5
1	3	LOC728946	XM_001128870	2	17	87345	87345	C	T	MEL9
1	3	LOC728946	XM_001128870	5	17	87355	87355	G	A	MEL4
1	0	LOC729025	XM_001129090	476	12	16261150	16261150	C	T	MEL11
1	0	LOC729025	XM_001129090	476	12	16261150	16261150	C	T	MEL8
1	4	LOC731932	XM_001716667	18	13	45933148	45933148	C	T	MEL6
1	4	LOC731932	XM_001716667	22	13	45933135	45933135	G	A	MEL9
1	7	MACROD2	NM_080676	418	20	15978497	15978497	G	C	MEL5
1	7	MACROD2	NM_080676	411	20	15978474	15978474	G	A	MEL5
1	4	MAPK10	NM_138982	414	4	87169385	87169385	G	A	MEL9
1	4	MAPK10	NM_138982	418	4	87157548	87157548	C	T	MEL4
1	0	MBD5	NM_018328.3	1350	2	148964418	148964418	C	T	H_LX-WU_MEL_521
1	0	MBD5	NM_018328.3	1350	2	148964419	148964419	C	T	H_LX-WU_MEL_521
1	0	MLL2	NM_003482.3	4945	12	47707182	47707182	G	A	H_LX-WU_MEL_522
1	0	MLL2	NM_003482.3	4945	12	47707183	47707183	G	C	H_LX-WU_MEL_522
1	4	MMACHC	NM_015506	117	1	45746543	45746543	G	A	MEL9
1	4	MMACHC	NM_015506	113	1	45746532	45746532	C	T	MEL6
1	9	MUC4	ENST00000405167	3838	3	196991334	196991334	C	T	H_LX-167
1	5	MUC4	ENST00000405167	3616	3	196992000	196992000	G	A	H_LX-167
1	9	MUC4	ENST00000405167	3174	3	196993324	196993325	0	G	H_LX-167
1	5	MUC4	ENST00000405167	3621	3	196991984	196991984	C	T	H_LX-167
1	9	MUC4	ENST00000405167	3829	3	196991360	196991360	T	C	H_LX-167
1	9	MUC4	ENST00000405167	3165	3	196993353	196993353	C	G	H_LX-WU_MEL_21
1	9	MUSK	NM_005592	520	9	112587089	112587089	A	T	MEL6
1	9	MUSK	NM_005592	529	9	112587627	112587627	G	A	MEL9

1	7	MYO7A	NM_000260	2039	11	76600024	76600024	G	A	MEL6
1	7	MYO7A	NM_000260	2032	11	76600001	76600001	C	T	MEL4
1	1	NCF2	NM_000433	187	1	181808993	181808993	C	T	MEL3
1	1	NCF2	NM_000433	188	1	181808989	181808989	C	T	MEL9
1	4	NIPBL	NM_133433	1670	5	37055260	37055260	G	A	MEL9
1	4	NIPBL	NM_133433	1674	5	37056327	37056327	A	G	MEL8
1	9	NPY1R	NM_000909	138	4	164466744	164466744	C	T	MEL9
1	9	NPY1R	NM_000909	147	4	164466717	164466717	C	G	MEL6
1	3	NRK	ENST00000243300	1325	X	105074600	105074600	G	A	H_LX-80
1	3	NRK	ENST00000243300	1322	X	105074592	105074592	A	C	MEL5
1	2	NUAK1	NM_014840	544	12	104985066	104985066	C	T	MEL7
1	2	NUAK1	NM_014840	546	12	104985059	104985059	G	A	MEL5
1	0	ODZ3	NM_001080477	858	4	183887315	183887315	C	T	MEL9
1	0	ODZ3	NM_001080477	858	4	183887315	183887315	C	T	MEL8
1	8	OR1S1	NM_001004458	85	11	57739046	57739046	C	T	MEL6
1	8	OR1S1	NM_001004458	93	11	57739070	57739070	A	T	MEL4
1	0	OR2A5	NM_012365	21	7	143378489	143378489	G	A	MEL6
1	0	OR2A5	NM_012365	21	7	143378489	143378489	G	A	MEL3
1	0	OR2AP1	ENST00000321688	171	12	54254977	54254977	G	A	MEL4
1	0	OR2AP1	ENST00000321688	171	12	54254977	54254977	G	A	MEL11
1	0	OR2T4	NM_001004696.1	253	1	246592262	246592262	A	0	H_LX-167
1	0	OR2T4	NM_001004696.1	253	1	246592262	246592262	A	0	H_LX-174
1	1	OR4A13P	ENST00000314689	23	11	54991174	54991174	T	0	H_LX-187
1	1	OR4A13P	ENST00000314689	24	11	54991177	54991177	G	A	MEL9
1	4	OR4P4	NM_001004124	273	11	55163226	55163226	T	A	MEL6
1	4	OR4P4	NM_001004124	277	11	55163239	55163239	C	T	MEL2
1	1	OR52B4	NM_001005161	244	11	4345371	4345371	C	T	MEL9
1	1	OR52B4	NM_001005161	245	11	4345368	4345368	G	A	MEL3
1	2	PAPPA2	NM_020318.2	1242	1	174947666	174947666	G	A	H_LX-WU_ Mel_218
1	2	PAPPA2	NM_020318.2	1244	1	174947672	174947672	G	A	H_LX-80
1	4	PCDH15	ENST00000395445	1733	10	55238617	55238617	T	A	MEL10
1	4	PCDH15	ENST00000395445	1737	10	55238607	55238607	C	T	MEL9
1	6	PCDH15	NM_033056	843	10	55450181	55450181	G	A	MEL9
1	6	PCDH15	NM_033056	849	10	55450164	55450164	C	T	MEL4
1	5	PCLO	NM_033026	1302	7	82433135	82433135	G	A	MEL9
1	5	PCLO	NM_033026	1307	7	82433121	82433121	G	A	MEL9
1	4	PCSK5	ENST00000376754	1040	9	78126282	78126282	G	A	MEL6
1	4	PCSK5	ENST00000376754	1044	9	78126294	78126294	G	A	MEL5
1	10	PENK	NM_006211	219	8	57516534	57516534	G	A	MEL9
1	10	PENK	NM_006211	209	8	57516563	57516563	C	T	MEL2
1	5	PIWIL1	NM_004764	75	12	129396284	129396284	C	T	MEL6
1	5	PIWIL1	NM_004764	70	12	129396269	129396269	G	A	MEL9
1	2	PKHD1	NM_138694	3776	6	51620859	51620859	G	A	MEL1
1	2	PKHD1	NM_138694	3778	6	51620853	51620853	C	T	MEL9
1	3	PLCZ1	NM_033123	476	12	18739144	18739144	T	C	MEL5
1	3	PLCZ1	NM_033123	479	12	18739137	18739137	C	T	MEL11
1	1	POTEA	NM_001005365	311	8	43290217	43290217	G	C	MEL11
1	1	POTEA	NM_001005365	310	8	43290214	43290214	G	A	MEL6
1	3	PPP1R3A	NM_002711	486	7	113306926	113306926	C	T	MEL11

1	3	PPP1R3A	NM_002711	483	7	113306935	113306935	C	T	MEL7
1	8	PREX2	ENST00000396539	898	8	69178426	69178426	C	T	H_LX-80
1	8	PREX2	ENST00000396539	890	8	69178396	69178396	C	T	H_LX-WU_ MEL_522
1	8	PTHLH	NM_198965	94	12	28007791	28007791	C	T	MEL4
1	8	PTHLH	NM_198965	102	12	28007767	28007767	C	T	MEL6
1	1	PTPRR	NM_002849.2	478	12	69364239	69364239	G	A	H_LX-WU_ Mel_218
1	1	PTPRR	NM_002849.2	477	12	69364240	69364240	C	G	H_LX-WU_ Mel_218
1	4	PTPRR	NM_002849	334	12	69425872	69425872	G	A	MEL6
1	4	PTPRR	NM_002849	338	12	69381366	69381366	C	T	MEL9
1	9	RASA1	NM_002890	481	5	86694232	86694232	C	T	MEL9
1	9	RASA1	NM_002890	472	5	86694205	86694205	T	C	MEL2
1	2	RNF180	ENST00000389100	463	5	63656928	63656928	G	A	MEL11
1	2	RNF180	ENST00000389100	465	5	63656934	63656934	C	T	MEL4
1	6	ROBO3	NM_022370	329	11	124245786	124245786	G	A	MEL13
1	5	ROBO3	NM_022370	380	11	124246226	124246226	G	A	MEL9
1	6	ROBO3	NM_022370	335	11	124245805	124245805	C	T	MEL9
1	5	ROBO3	NM_022370	375	11	124246210	124246210	C	T	MEL5
1	2	SAGE1	NM_018666	197	X	134815983	134815983	G	A	MEL6
1	2	SAGE1	NM_018666	195	X	134815978	134815978	C	T	MEL8
1	7	SALL1	NM_002968	76	16	49733408	49733408	C	T	MEL11
1	4	SALL1	NM_002968	391	16	49732463	49732463	G	A	MEL9
1	7	SALL1	NM_002968	83	16	49733387	49733387	C	T	MEL1
1	4	SALL1	NM_002968	387	16	49732474	49732474	G	A	MEL9
1	0	SCN8A	NM_014191	1638	12	50486449	50486449	C	T	MEL6
1	0	SCN8A	NM_014191	1638	12	50486449	50486449	C	T	MEL11
1	3	SELPLG	NM_003006	107	12	107541893	107541893	G	A	MEL9
1	3	SELPLG	NM_003006	110	12	107541884	107541884	C	T	MEL6
1	10	SERPINB3	NM_006919	250	18	59475065	59475065	C	T	MEL3
1	10	SERPINB3	NM_006919	260	18	59474264	59474264	T	A	MEL10
1	4	SHC3	NM_016848	460	9	90843006	90843006	G	A	MEL9
1	4	SHC3	NM_016848	464	9	90842994	90842994	G	A	MEL6
1	3	SLAMF6	NM_052931	70	1	158732648	158732648	G	A	MEL12
1	3	SLAMF6	NM_052931	67	1	158732657	158732657	G	A	MEL9
1	0	SLC2A2	NM_000340	84	3	172215073	172215073	C	T	MEL12
1	0	SLC2A2	NM_000340	84	3	172215073	172215073	C	T	MEL6
1	10	SLC44A3	NM_152369	178	1	95078098	95078098	C	T	MEL4
1	10	SLC44A3	NM_152369	168	1	95075991	95075991	C	T	MEL8
1	2	SLC5A12	NM_178498	492	11	26659177	26659177	C	T	MEL9
1	2	SLC5A12	NM_178498	494	11	26656933	26656933	C	T	MEL4
1	8	SLC9A4	NM_001011552	662	2	102509184	102509184	C	T	MEL12
1	8	SLC9A4	NM_001011552	670	2	102509208	102509208	G	A	MEL5
1	9	SLCO6A1	NM_173488	37	5	101862338	101862338	C	T	MEL6
1	9	SLCO6A1	NM_173488	46	5	101862311	101862311	T	C	MEL11
1	4	SPTB	NM_001024858	1367	14	64318928	64318928	T	G	MEL3
1	4	SPTB	NM_001024858	1363	14	64318940	64318940	C	T	MEL13
1	0	STAB2	NM_017564	2340	12	102676108	102676108	C	T	MEL6
1	0	STAB2	NM_017564	2340	12	102676108	102676108	C	T	MEL5

1	0	STAC	NM_003149	186	3	36501539	36501539	G	A	MEL6
1	0	STAC	NM_003149	186	3	36501539	36501539	G	A	MEL3
1	4	SULT1B1	NM_014465	35	4	70655422	70655422	G	A	MEL9
1	4	SULT1B1	NM_014465	39	4	70655409	70655409	T	C	MEL2
1	0	TBX15	ENST00000393149	248	1	119229121	119229121	G	A	MEL1
1	0	TBX15	ENST00000393149	248	1	119229121	119229121	G	A	MEL11
1	6	TGFBI	NM_000358	507	5	135419376	135419376	G	A	MEL6
1	6	TGFBI	NM_000358	501	5	135419358	135419358	C	T	MEL6
1	10	TGM3	NM_003245	305	20	2246062	2246062	G	A	MEL1
1	10	TGM3	NM_003245	315	20	2246091	2246091	C	A	MEL3
1	3	THSD7B	NM_001080427	126	2	137530789	137530789	G	A	MEL7
1	3	THSD7B	NM_001080427	129	2	137530798	137530798	G	A	MEL5
1	1	TMPRSS11B	NM_182502	375	4	68776351	68776351	G	A	MEL9
1	1	TMPRSS11B	NM_182502	374	4	68776355	68776355	C	T	MEL7
1	1	TMPRSS7	NM_001042575	379	3	113277023	113277023	G	A	MEL4
1	1	TMPRSS7	NM_001042575	378	3	113277019	113277019	C	T	MEL12
1	0	TNRC6B	NM_015088.1	169	22	38990686	38990686	G	A	H_LX-167
1	0	TNRC6B	NM_015088.1	169	22	38990687	38990687	G	A	H_LX-167
1	2	TRHDE	NM_013381	761	12	71299032	71299032	C	T	MEL11
1	2	TRHDE	NM_013381	763	12	71299039	71299039	C	T	MEL6
1	0	TRIM46	NM_025058	703	1	153423118	153423118	C	T	MEL7
1	0	TRIM46	NM_025058	703	1	153423118	153423118	C	T	MEL13
1	4	TRPC6	NM_004621	421	11	100864910	100864910	C	T	MEL9
1	4	TRPC6	NM_004621	425	11	100864897	100864897	C	T	MEL6
1	6	uc003vxc.2	ENST00000304182	144	7	141601358	141601358	C	T	MEL4
1	6	uc003vxc.2	ENST00000304182	150	7	141598898	141598898	C	T	MEL6
1	9	uc003vyi.2	ENST00000390365	17	7	141776303	141776303	G	A	MEL9
1	9	uc003vyi.2	ENST00000390365	26	7	141776331	141776331	C	T	MEL9
1	4	uc003vza.1	ENST00000390381	82	7	141909061	141909061	G	A	MEL6
1	4	uc003vza.1	ENST00000390381	86	7	141909073	141909073	G	A	MEL4
1	3	UNC13C	NM_001080534	1326	15	52373544	52373544	G	A	MEL8
1	3	UNC13C	NM_001080534	1323	15	52373535	52373535	G	A	MEL5
1	9	ZFHX4	NM_024721	1072	8	77853198	77853198	T	A	MEL6
1	9	ZFHX4	NM_024721	1063	8	77853171	77853171	C	T	MEL11
1	5	ZNF208	NM_007153	57	19	21961915	21961915	C	T	MEL6
1	5	ZNF208	NM_007153	62	19	21961899	21961899	G	A	MEL9
1	5	ZNF230	NM_006300	279	19	49206866	49206866	C	T	MEL4
1	5	ZNF230	NM_006300	274	19	49206851	49206851	C	T	MEL9
1	4	ZNF343	NM_024325	555	20	2411944	2411944	C	T	MEL6
1	4	ZNF343	NM_024325	559	20	2411931	2411931	C	T	MEL9
1	3	ZNF713	NM_182633	272	7	55974715	55974715	C	T	MEL5
1	3	ZNF713	NM_182633	269	7	55974706	55974706	G	A	MEL6
1	9	ZNF725	ENST00000334589	101	19	23466925	23466925	G	A	MEL9
1	9	ZNF725	ENST00000334589	92	19	23479819	23479819	C	T	MEL11
1	0	ZNF831	NM_178457	1393	20	57261578	57261578	G	A	MEL6
1	0	ZNF831	NM_178457	1393	20	57261578	57261578	G	A	MEL7

“Mutations within proximity” shows the number of mutations on the same transcript within 10 amino acids and the “nearest mutations” show the number of amino acid between the closest mutations. By our definition, if more than 1 mutation occurs in the same amino acid, the distance to the closest mutation would be “0” amino acids.

Supplementary Table S2: Characteristics of metastatic melanoma samples analyzed in Figure 3

A

	ALL (*n* = 253)				
	RASA1 Low (*n* = 126)			RASA1 High (*n* = 127)	
**Variables**	**Frequency**	**%**		**Frequency**	**%**
**Age**					
[18, 40]	18	14.3		23	18.1
(40, 60]	49	38.9		57	44.9
(60, 100]	59	46.8		47	37.0
**Gender**					
Female	53	42.1		43	33.9
Male	73	57.9		84	66.1
**Tumor Type**					
Cutaneous	122	96.8		124	97.6
Mucosal	2	1.6		2	1.6
Ocular	2	1.6		1	0.8
**Metastasis location**					
Brain	15	11.9		16	12.6
Lung	10	7.9		14	11.0
Lymph nodes	54	42.9		54	42.5
Skin/soft tissue	30	23.8		29	22.8
Others	17	13.5		14	11.0

B

	BRAF wild-type (*n* = 61)				BRAF mutant (*n* = 42)			
	RASA1 Low (*n* = 30)		RASA1 High (*n* = 31)		RASA1 Low (*n* = 21)		RASA1 High (*n* = 21)	
**Variables**	**Frequency**	**%**	**Frequency**	**%**	**Frequency**	**%**	**Frequency**	**%**
**Age**								
[18, 40]	5	16.7	5	16.1	2	9.5	4	19.
(40, 60]	13	43.3	14	45.2	11	52.4	14	66.7
(60, 100]	12	40.0	12	38.7	8	38.1	3	14.3
**Gender**								
Female	14	46.7	8	25.8	7	33.3	10	47.6
Male	16	53.3	23	74.2	14	66.7	11	52.4
**Tumor Type**								
Cutaneous	30	100.0	30	96.8	21	100.0	21	100.0
Mucosal	0	0.0	1	3.2	0	0.0	0	0.0
Ocular	0	0.0	0	0.0	0	0.0	0	0.0

**Metastasis location**								
Brain	4	13.3	2	6.5	3	14.3	1	4.8
Lung	5	16.7	4	12.9	0	0.0	4	19.0
Lymph nodes	6	20.0	12	38.7	11	52.4	8	38.1
Skin/soft tissue	7	23.3	7	22.6	6	28.6	5	23.8
Others	8	26.7	6	19.4	1	4.8	3	14.3

Age of diagnosis for primary melanoma, gender, location of metastasis, and histological types are shown for each groups of samples.
